# Cell-Free Expression of Sodium Channel Domains for Pharmacology Studies. Noncanonical Spider Toxin Binding Site in the Second Voltage-Sensing Domain of Human Na_v_1.4 Channel

**DOI:** 10.3389/fphar.2019.00953

**Published:** 2019-09-04

**Authors:** Mikhail Yu. Myshkin, Roope Männikkö, Olesya A. Krumkacheva, Dmitrii S. Kulbatskii, Anton O. Chugunov, Antonina A. Berkut, Alexander S. Paramonov, Mikhail A. Shulepko, Matvey V. Fedin, Michael G. Hanna, Dimitri M. Kullmann, Elena G. Bagryanskaya, Alexander S. Arseniev, Mikhail P. Kirpichnikov, Ekaterina N. Lyukmanova, Alexander A. Vassilevski, Zakhar O. Shenkarev

**Affiliations:** ^1^Shemyakin and Ovchinnikov Institute of Bioorganic Chemistry, Russian Academy of Sciences, Moscow, Russia; ^2^MRC Centre for Neuromuscular Diseases, Department of Molecular Neuroscience, UCL Institute of Neurology, London, United Kingdom; ^3^International Tomography Center SB RAS, Novosibirsk, Russia; ^4^School of Biological and Medical Physics, Moscow Institute of Physics and Technology (State University), Dolgoprudny, Russia; ^5^International Laboratory for Supercomputer Atomistic Modelling and Multi-scale Analysis, National Research University Higher School of Economics, Moscow, Russia; ^6^Department of Clinical and Experimental Epilepsy, UCL Institute of Neurology, London, United Kingdom; ^7^N.N.Voroztsov Novosibirsk Institute of Organic Chemistry SB RAS, Novosibirsk, Russia; ^8^Faculty of Biology, Lomonosov Moscow State University, Moscow, Russia

**Keywords:** channelopathies, sodium channel, gating modifier, NMR spectroscopy, cell-free expression, combinatorial selective labeling, ligand-receptor interactions

## Abstract

Voltage-gated sodium (Na_V_) channels are essential for the normal functioning of cardiovascular, muscular, and nervous systems. These channels have modular organization; the central pore domain allows current flow and provides ion selectivity, whereas four peripherally located voltage-sensing domains (VSDs-I/IV) are needed for voltage-dependent gating. Mutations in the S4 voltage-sensing segments of VSDs in the skeletal muscle channel Na_V_1.4 trigger leak (gating pore) currents and cause hypokalemic and normokalemic periodic paralyses. Previously, we have shown that the gating modifier toxin Hm-3 from the crab spider *Heriaeus melloteei* binds to the S3-S4 extracellular loop in VSD-I of Na_V_1.4 channel and inhibits gating pore currents through the channel with mutations in VSD-I. Here, we report that Hm-3 also inhibits gating pore currents through the same channel with the R675G mutation in VSD-II. To investigate the molecular basis of Hm-3 interaction with VSD-II, we produced the corresponding 554-696 fragment of Na_V_1.4 in a continuous exchange cell-free expression system based on the *Escherichia coli* S30 extract. We then performed a combined nuclear magnetic resonance (NMR) and electron paramagnetic resonance spectroscopy study of isolated VSD-II in zwitterionic dodecylphosphocholine/lauryldimethylamine-N-oxide or dodecylphosphocholine micelles. To speed up the assignment of backbone resonances, five selectively ^13^C,^15^N-labeled VSD-II samples were produced in accordance with specially calculated combinatorial scheme. This labeling approach provides assignment for ∼50% of the backbone. Obtained NMR and electron paramagnetic resonance data revealed correct secondary structure, quasi-native VSD-II fold, and enhanced ps–ns timescale dynamics in the micelle-solubilized domain. We modeled the structure of the VSD-II/Hm-3 complex by protein–protein docking involving binding surfaces mapped by NMR. Hm-3 binds to VSDs I and II using different modes. In VSD-II, the protruding ß-hairpin of Hm-3 interacts with the S1-S2 extracellular loop, and the complex is stabilized by ionic interactions between the positively charged toxin residue K24 and the negatively charged channel residues E604 or D607. We suggest that Hm-3 binding to these charged groups inhibits voltage sensor transition to the activated state and blocks the depolarization-activated gating pore currents. Our results indicate that spider toxins represent a useful hit for periodic paralyses therapy development and may have multiple structurally different binding sites within one Na_V_ molecule.

## Introduction

**Figure 1:**
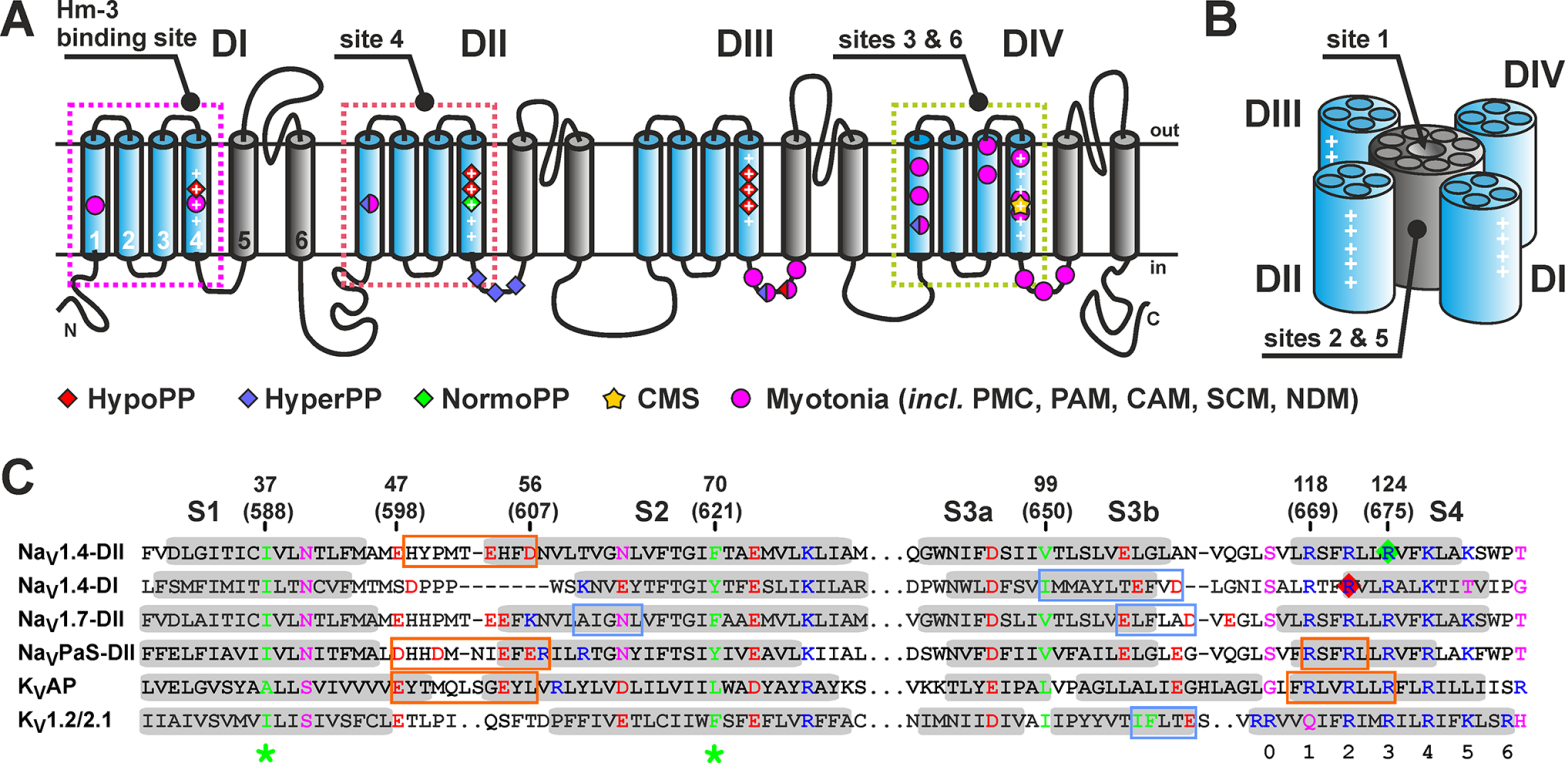
**(A)** Transmembrane topology of Na_V_ channels. The S1-S4 helices are in blue and S5-S6 in gray. Conserved Arg/Lys residues are marked by the plus sign (+). Sites of the VSD mutations associated with different diseases (Simkin, 2011; Nicole and Fontaine, 2015) are marked (HypoPP, NormoPP, HyperPP, hypokalemic, normokalemic, or hyperkalemic periodic paralysis; CMS, congenital myasthenic syndrome). **(B)** Spatial organization of Na_V_ channels with one pore domain and four VSDs. Approximate positions of the ligand-binding sites are shown. **(C)** Alignment of VSD-II and VSD-I of human Na_V_1.4 channel with VSDs of other Na_V_ and K_V_ channels. Conserved aromatic/hydrophobic, charged, and polar residues are color-coded. TM segments are highlighted in gray. The gating charge transfer center is marked by green asterisks. The sites of conserved charged residues in the S4 helix are numbered. Mutations of R222 (Na_V_1.4-DI) and R675 (Na_V_1.4-DII) (red and green diamonds) are associated with HypoPP and NormoPP, respectively. The binding sites of spider GMTs are boxed. There are two types of sites with the primary binding interface on the S3b helix (blue) and on the S1-S2 loop (orange). Sites are shown for: Na_V_1.4-DII/Hm-3 (present work); Na_V_1.4-DI/Hm-3 (Männikkö et al., 2018); Na_V_1.7-DII/ProTx2 (Xu et al., 2019); Na_V_PaS-DII/Dc1a (Shen et al., 2018); K_V_AP/VsTx1 (Lau et al., 2016); and K_V_1.2-2.1/hanatoxin (Swartz and MacKinnon, 1997). Two numbering schemes are given: residue numbers in the expressed VSD-II construct starting from the *N*-terminal Met1 and in the full-length Na_V_1.4 channel (in parentheses).

Voltage-gated sodium channels (Na_V_) together with potassium and calcium channels form a large P-loop superfamily of integral membrane proteins (IMPs) (Hille, 2001). α-Subunits of eukaryotic Na_V_ channels have modular architecture and are composed of four homologous repeats (**Figure 1A**), each closely related to a subunit of homotetrameric voltage-gated potassium channels (K_V_) or bacterial Na_V_ channels. Every repeat consists of six transmembrane (TM) α-helices (S1-S6) and contains a voltage-sensing domain (VSD) formed by helices S1-S4. The central pore and selectivity filter are formed by helices S5-S6 from all four repeats (**Figure 1B**) (Catterall, 2014). The S4 helix of the VSDs, sometimes called the “voltage sensor,” accommodates several positively charged groups (usually four Arg residues, R1-4) referred to as gating charges, which reposition relative to a hydrophobic gating charge transfer center (formed by conserved aromatic/hydrophobic residues in the middle of S1 and S2 helices, **Figure 1C**) when the transmembrane voltage changes (Tao et al., 2010; Henrion et al., 2012; Jensen et al., 2012). At the resting voltage, the S4 segment is in the “down” state but moves to the “up” state upon depolarization, exposing gating charges to the extracellular environment.

Na_V_ channels carry the key depolarizing current of the action potential that controls a wide range of physiological phenomena, including the excitability of cardiac, muscle, and neuronal cells and propagation of nerve signals (Hille, 2001). There is a variety of neurological, cardiovascular, and neuromuscular disorders caused by malfunctions of these channels (Ashcroft, 2000). For example, mutations of conserved S4 arginine residues in the skeletal muscle Na_V_1.4 channel (*SCN4A*) trigger leak currents through the VSDs, known as “gating pore currents” or “ω-currents” that are induced in addition to the main pore or α-currents (Groome et al., 2018). Gating pore currents constitute the pathomechanism of several diseases such as hypokalemic and normokalemic periodic paralyses (HypoPP or NormoPP) (**Figure 1A**) (Matthews et al., 2009; Suetterlin et al., 2014). An important feature of gating pore currents is their voltage dependence, which relates to the position of the mutated arginine residue. Mutation of the outer R1 or R2 residues leads to gating pore currents that are activated at resting potentials or hyperpolarization (Sokolov et al., 2007; Struyk and Cannon, 2007; Struyk et al., 2008), whereas replacements of the inner R3 residues induce depolarization-activated currents (Sokolov et al., 2008).

VSDs of different channels, as well as four VSDs within one Na_V_ channel (VSDs I-IV), demonstrate marked structural variability and frequently possess unique ligand binding sites (Stevens et al., 2011). This makes them attractive targets for the development of new selective drugs against various diseases associated with channel dysfunction (channelopathies) (Li et al., 2013; Ahuja et al., 2015). Recently, we have shown that a gating modifier toxin (GMT) Hm-3 from the crab spider *Heriaeus melloteei* can inhibit gating pore currents due to mutations affecting the second or third arginine residue (R2 or R3; R222G/W or R225G, respectively) in the S4 helix of VSD-I of Na_V_1.4 channel; such R2 mutations were found in patients with HypoPP (Männikkö et al., 2018). Curiously, the toxin did not affect gating pore currents through R1 (R219G) mutant channels. And, more importantly, it did not modify currents through VSDs II or III with R2 replacements (R672G and R1132Q). Selective inhibition of gating pore currents through domain I by Hm-3 correlated perfectly with its ability to suppress currents through chimeric K_V_2.1 channels with the extracellular facing parts of the S3-S4 helices (so-called “paddle motif”) transferred from VSD-I of Na_V_1.4 but not through chimeric channels with the insertion of paddle motifs from three other VSDs. We concluded that Hm-3 is a selective VSD-I toxin (Männikkö et al., 2018). Thus, Hm-3 and similar GMTs interacting with VSDs may constitute useful hits in developing gating pore current inhibitors and HypoPP therapy.

To enable development of GMTs or GMT-like compounds toward selective gating pore current blockers, it is imperative to describe the molecular interaction of the toxins with the channel. However, one of the main problems hampering structural studies of ion channels is large-scale production of IMPs in folded and functional state. In recent years, cell-free (CF) systems have attracted increasing attention as alternative tools for recombinant production of IMPs and their hydrophobic domains (Klammt et al., 2006; Henrich et al., 2015). The full power of CF expression becomes apparent in nuclear magnetic resonance (NMR) studies because they allow producing proteins with different patterns of selective isotope (^2^H, ^13^C, ^15^N) labeling (Kainosho et al., 2006; Reckel et al., 2011). There are two general approaches to CF production of IMPs: in soluble form in the presence of membrane-mimicking components (detergents, lipids, nanodiscs, etc.) and in insoluble form in the absence of any membrane mimetics (Lyukmanova et al., 2012; Henrich et al., 2015). Insoluble IMPs require further solubilization by an appropriate detergent.

Previously, CF synthesis in the form of insoluble precipitate allowed us to produce milligram quantities of several variants of isotopically labeled VSD-I of Na_V_1.4 channel (isolated S1-S4 domain, 134 residues) and to study its complex with Hm-3 toxin by NMR (Männikkö et al., 2018). Our data revealed toxin binding to the outer fragment of the S3 helix (S3b) and extracellular S3-S4 loop. Such mode of spider toxin binding was later confirmed by crystallographic and cryo-electron microscopy studies of chimeric Na_V_Ab/hNa_V_1.7 channel in complex with ProTx2 toxin (Xu et al., 2019). However, structural studies of Dc1a and VsTx1 toxins in complex with Na_V_PaS and K_V_AP channels, respectively (Lau et al., 2016; Shen et al., 2018), revealed that there is an alternative GMT binding site located on the S1-S2 loop of the VSDs (**Figure 1C**).

In this report, we show that, surprisingly, Hm-3 toxin inhibits gating pore currents, which occur due to the R675G mutation of the third arginine residue (R3) in the S4 helix of VSD-II of the Na_V_1.4 channel. This mutation was found in patients with NormoPP (Vicart et al., 2004). CF expression system provided sufficient quantities of differently labeled variants of isolated VSD-II and allowed us to characterize the structure and dynamics of VSD-II by NMR and electron paramagnetic resonance (EPR) spectroscopy. Our NMR study of the VSD-II/Hm-3 complex reveals that the toxin binds to VSD-II in a different mode compared with VSD-I.

## Materials and Methods

### Hm-3 and VsTx1 Production

Hm-3 and ^15^N-labeled Hm-3 were produced recombinantly according to published protocols (Berkut et al., 2015; Männikkö et al., 2018). Briefly, the peptide-encoding nucleotide sequence was cloned into the pET-32b vector (Novagen), and Hm-3 was synthesized as part of a fusion protein with thioredoxin (Trx). The His-tagged Trx-Hm-3 fusion protein was isolated by Co^2+^-chromatography and cleaved by cyanogen bromide according to published protocol (Andreev et al., 2010); Hm-3 was purified by reversed-phase high-performance liquid chromatography. For ^15^N-labeled Hm-3, the minimal growth medium M9 was used containing 0.1% ^15^NH_4_Cl. VsTx1 analogue was produced according to published protocol (Shenkarev et al., 2014) as part of a fusion protein with Trx. Obtained toxin analogue contained additional N-terminal Gly-Ser residues resulting from the hydrolysis of the fusion construct by thrombin. ^15^N-labeled VsTx1 was produced similarly to the labeled Hm-3.

### Electrophysiology

Gating pore currents through mutant Na_V_1.4 channels were studied using two-electrode voltage clamp in *Xenopus* oocytes. All procedures were carried out as described previously (Männikkö et al., 2018). Briefly, oocytes for Na_V_1.4 expression were isolated from *Xenopus laevis* in accordance with the UK Animal (Scientific Procedures) Act 1986. The oocytes were injected with rat *SCN4A* encoding R669G mutant channel (analogous to human R675G mutation) and *SCN1B* messenger RNA transcribed *in vitro* (mMESSAGE mMACHINE kit, Thermo Fisher Scientific) at a 1:1 mass ratio using Nanoject (Drummond). GeneClamp 500B, Digidata 1200, and pCLAMP programs (Molecular Devices) were used to collect data. The bath solution contained 60-mM sodium methanesulfonate, 60-mM guanidine sulfate, 1.8-mM CaSO_4_, and 10-mM Hepes (pH 7.4), and the oocytes were perfused with 1–2-μM tetrodotoxin (TTX) to block the main pore. To measure gating pore currents, the mean current during the last 100 ms of a 300-ms step to voltages ranging from −140 mV to +50 mV in 5-mV increments was plotted against the voltage. Holding voltage was -100 mV. Data were analyzed and presented using pCLAMP (Molecular Devices) and Origin (OriginLab) software. All data are presented as mean ± SEM.

### Cell-Free Production of Voltage-Sensing Domain II Samples

Second VSD of the human Na_V_1.4 channel (residues 554-696) containing C660S, C661S, and C687S mutations and additional *N*-terminal Met-Gly and *C*-terminal Gly-Ser-His_6_ fragments were produced as described in Paramonov et al. (2017) (final protein sequence is presented in **Figure 5**). Briefly, corresponding gene with codons optimized for *Escherichia coli* was cloned into the pIVEX2.3d vector (Roche) and used as a template in continuous exchange CF expression system based on the *E. coli* S30 extract as described in Lyukmanova et al. (2012). The volume ratio of the reaction mixture (RM) to feeding mixture (FM) was 1:15. The dialysis membrane tubing with the cutoff of 12 kDa (Sigma Aldrich) was used to separate the RM and FM. CF reactions were performed without addition of any membrane-mimicking components into the RM. The synthesis was carried out at 30°C with gentle mixing for 20 h. Soluble and insoluble fractions of the RM after synthesis were separated by centrifugation for 15 min at 14,000 rpm. The RNA and DNA traces were removed as described in Paramonov et al. (2017).

^15^N-Labeled and ^13^C,^15^N-labeled VSD-II samples were synthesized using ^15^N or ^13^C,^15^N algal amino acid (AA) mixture (Isotec, Sigma-Aldrich) at a concentration of 3.7 mg/ml. ^15^N- or ^13^C,^15^N-Trp, ^15^N- or ^13^C,^15^N-Gln, and ^15^N-Asn (CIL) were added to the RM and FM at concentrations of 2.3, 1.3, and 1.3 mM, respectively. For production of selectively labeled samples, individual non-labeled (Sigma-Aldrich), and ^13^C’-, ^15^N- or ^13^C,^15^N-labeled AAs (CIL) were used. Concentrations of the individual AAs were 1 mM each. The yield of the isotope-labeled VSD-II was ∼1 mg of the target protein from 1 ml of RM.

### Preparation of Samples for Nuclear Magnetic Resonance and Electron Paramagnetic Resonance Spectroscopy

Precipitate from 1 to 1.5 ml of the RM was solubilized in 70 µl of buffer A (20-mM Tris-HCl, 300-mM NaCl, pH 8.0) containing 3.5% (100 mM) n-dodecylphosphocholine (DPC, FOS-12, Anatrace) and diluted to 500 µl by buffer A. The samples were purified by Ni^2+^-chromatography in the presence of 0.5% (14 mM) DPC. Fractions with VSD-II were eluted by buffer A containing 500-mM imidazole and 0.5% DPC. The buffer in the samples was changed to buffer B (20-mM Tris-Ac, 1-mM sodium azide, 5% D_2_O, pH 5.5) by four repeated cycles of dilution/concentration using Stirred Cells with regenerated nitrocellulose Ultrafiltration Membranes, NMWL 10,000 (Millipore). The samples were concentrated to 350 μl. Final DPC concentration was 10–35 mM, and n-dodecyl-N,N-dimethylamine-N-oxide (LDAO, Anatrace) was added to an equal molar ratio. Concentrations of the detergents in the samples were measured by one-dimensional (1D) ^1^H NMR spectroscopy.

For preparation of the single and double spin-labeled samples, three VSD-II mutants (A45C, M25C/S115C, and A45C/S131C, the residue numbers correspond to the expressed VSD construct starting from Met1) were produced using a CF system as described previously. The protein precipitates were solubilized in buffer A containing 3.5% DPC or 1-palmitoyl-2-hydroxy-sn-glycero-3-[phospho-rac-(1-glycerol)] (LPPG, Avanti Polar Lipids) and incubated with 2-mM dithiothreitol (DTT) during 20 min at 30°C, and DTT was removed by size-exclusion chromatography (SEC) on a NAP-5 column (GE Healthcare). After that, 2.5 mM S-(1-oxyl-2,2,5,5-tetramethyl-2,5-dihydro-1H-pyrrol-3-yl)methyl methanesulfonothioate (MTSL, Toronto Research Chemicals) was added followed by 20-min incubation at 30°C and overnight incubation at 23°C. The samples were purified by Ni^2+^-chromatography in the presence of 0.5% DPC or LPPG.

To remove protein aggregates, the spin-labeled VSD-II samples were additionally purified by SEC using a Tricorn 10/300 column prepacked with Superdex 200 pg (GE Healthcare) in 20-mM Tris-HCl, 150-mM NaCl, pH 5.5, 0.3% DPC. Ferritin [molecular weight (MW) 440 kDa, R_St_ 6.10 nm], catalase (MW 232 kDa, R_St_ 5.22 nm), aldolase (MW 158 kDa, R_St_ 4.81 nm), BSA (MW 67 kDa, R_St_ 3.55 nm), and ovalbumin (MW 43 kDa, R_St_ 3.05 nm) from high and low weight calibration kits (GE Healthcare) were used for calibration. The elution rate was 0.3 ml/min; the wavelength of detection was 280 nm. The measured elution volumes were converted to R_St_ values *via* a linear calibration graph [elution volume versus Log(R_St_)].

The buffer in the samples was changed to buffer B prepared on 100% D_2_O and containing 0.1% *d* *_38_*-DPC (CIL) or LPPG using four cycles of dilution/concentration on Stirred Cells with regenerated nitrocellulose Ultrafiltration Membranes, NMWL 30,000 (Millipore) and following concentration on 10-kDa Amicon Ultra-0.5 mL Centrifugal Filters (Millipore) to the volume of 50–100 μl. Final protein concentration was ∼200 μM, and *d* *_38_*-DPC or LPPG concentrations were 5–6%. For EPR measurements, *d* *_38_*-DPC or LPPG was added to the samples to the final detergent concentration of 15–40%.

### Nuclear Magnetic Resonance Spectroscopy

NMR spectra were measured at 45°C on Bruker Avance-III 600 and Avance-III 800 spectrometers, equipped with cryoprobes. TROSY-based 3D HNCO, HNCA, HN(CO)CA, and CBCACONH spectra and 3D NOESY-^15^N-HSQC spectrum (τ_m_ = 150 ms) were acquired at 45°C using 130-µM sample of uniformly ^13^C,^15^N-labeled VSD-II in buffer B containing DPC/LDAO micelles (35/35 mM). Non-uniform sampling method with 50% of sparse sampling was used. For the selectively labeled samples (concentrations of 50–80 µM), 2D ^1^H,^15^N-TROSY, and 2D ^1^H,^15^N-plans from HNCO-TROSY and HNCA-TROSY were acquired. The acquisition times were 1 h for TROSY and 5 h for 2D HNCO or HNCA spectra. ^1^H chemical shifts were referenced relative to the residual protons of H_2_O, the chemical shift of the signal being arbitrary chosen as 4.55 ppm at 45°C. For ^13^C and ^15^N nuclei, the indirect reference was used. Heteronuclear ^15^N-{^1^H} NOEs were measured for 57 non-overlapped and non-broadened ^15^NH groups at 80 MHz using standard experiment. Spectra were processed with MddNMR (Kazimierczuk and Orekhov, 2011) and TopSpin (Bruker) and analyzed in CARA program. Secondary chemical shifts were calculated by the TALOS-N software (Shen and Bax, 2013).

VSD-II/Hm-3 titrations were done in buffer B containing DPC/LDAO micelles (11/11 or 57/57 mM). Equilibrium dissociation constant of the VSD-II/Hm-3 complex (*K* *_V_*) was determined from the chemical shift titration data using a previously proposed method (Männikkö et al., 2018) with slight modification. The fast (at the NMR timescale) exchange of the Hm-3 molecules between three different states (free in solution, bound to the micelle and bound to VSD-II within the micelle) was assumed. To account for the presence of the two toxin binding sites on the domain, the Langmuir methodology was used. It was assumed that each empty micelle and VSD-II within the micelle contains two equivalent toxin binding sites formed by *N* *_SITE_* = 35 detergent molecules ([Micellar site]_0_ = [Detergent]_0_/*N* *_SITE_*). Thus, the VSD-II/micelle and VSD-II/Hm-3/micelle complexes contain equal amounts (*N* *_MIC_* = 70) of detergent molecules. The previously obtained data on the Hm-3 binding to DPC/LDAO (1:1) micelles (Männikkö et al., 2018) were refitted using the equation:

(Eq. 1). $$K_{M}[\mathrm{Hm}-3/\mathrm{Micellar site}]={\left[\mathrm{Hm}-3\right]}_{\mathrm{free}}\cdot {\left[\mathrm{Micellar site}\right]}_{\mathrm{free}}$$

The determined equilibrium dissociation constant of the Hm-3/“Micellar site” complex (*K* *_M_* = 90 μM) was used in the analysis of the Hm-3/VSD-II binding. The titration data (**Figure 8D**) were approximated using a system of equations, one of which describes Hm-3 binding to “Micellar site” (Eq. 1), and the other:

(Eq. 2), $$K_{V}\cdot [\mathrm{Hm}-3\text{/}\mathrm{VSD}-\mathrm{II}]=2\cdot {\left[\mathrm{Hm}-3\right]}_{\mathrm{free}}\cdot {\left[\mathrm{VSD}-\mathrm{II}\right]}_{\mathrm{free}}$$

describes Hm-3 binding to the sites on the VSD-II surface.

### Electron Paramagnetic Resonance Spectroscopy

Samples for EPR were placed in glass capillary tubes (outer diameter 1.5 mm, internal diameter 0.9 mm, with the sample volume being approximately 10 µl). Continuous wave (CW) EPR experiments were carried out at X-band (9 GHz) at 300 K and 140 K using a commercial Bruker EMX spectrometer. The experimental spectra were simulated using EasySpin (Stoll and Schweiger, 2006). Samples for double electron–electron resonance (DEER) measurements were prepared at room temperature, shock-frozen in liquid nitrogen, and studied at 50 K. The data were collected at a Q-band (34 GHz) Bruker Elexsys E580 pulse EPR spectrometer equipped with an EN5107D2 resonator and Oxford Instruments temperature control system (maximum available microwave power was limited to 1 W). A standard four-pulse DEER sequence (Pannier et al., 2000) was used with pulse lengths of 22/44 ns for probe (υ_probe_) and 44 ns for pump (υ_pump_) frequency and with two-step phase cycle. The time increment of the inversion pulse was 6 ns. The value of τ_1_ was 400 ns. The values of τ_2_ were 7,000 and 3,500 ns in *d* *_38_*-DPC and LPPG, respectively. The pump pulse was applied at the spectral maximum, and the measurements were done at the field position ∼2.2 mT higher than the maximum of the spectrum (Δυ = υ_pump_ − υ_probe_ = 60 MHz). All obtained DEER traces were background-corrected by exponential function and analyzed with Tikhonov regularization using DeerAnalysis2013 program (Jeschke et al., 2006). The actual form of the background function of the time-traces was determined by DEER measurements of the single-labeled A45-SL VSD-II variant. The function was found to be exponential with a fractal dimension D of 2.9. The maximal modulation depth for these DEER experimental parameters was ∼3%, which was verified using a short DNA duplex doubly labeled by nitroxides (Shevelev et al., 2015).

### Computer Modeling

Molecular dynamics (MD) simulation for the VSD-II (559-699 fragment of human Na_V_1.4 channel; PDB ID: 6AGF) was done in a hydrated three-component (palmitoyloleoylphosphatidylcholine, POPC; palmitoyloleoylphosphatidylethanolamine, POPE; cholesterol, CHOL; 2:1:1 molar ratio) bilayer. Receptor-in-the-membrane system was constructed with our in-house IMPULSE software, which permits semiautomatic construction, pre- and post-processing, and advanced analysis of complex MD systems. The system had a size of ∼11.1 × 11.1 × 12.6 nm^3^ and contained 234 POPC, 114 POPE, and 110 CHOL molecules; one VSD-II molecule; 33,549 H_2_O molecules; and one Na^+^ counter-ion that was added to maintain electroneutrality.

All simulations were performed with the GROMACS 5.1.2 suite (Abraham et al., 2015) using Amber99sb-ildn force field (Lindorff-Larsen et al., 2010) and TIP3P water model (González and Abascal, 2010). Other MD parameters were: semi-isotropic pressure of 1 bar and compressibility of 4.5 × 10^−5^ bar^−1^ (Berendsen barostat) and temperature of 37°C [V-rescale thermostat (Bussi et al., 2007)], PME electrostatics. Before MD production run, the system was energy-minimized, heated, and equilibrated in several stages. Heating was performed for 1 ns with VSD C^α^-atoms constrained to their positions to avoid structure deformation during the equilibration of lipids. Production MD run had a length of 200 ns with no fixed atoms.

Three MD trajectories of 200 ns each were calculated for Hm-3 in water. The conformational clustering of the VSD-II and Hm-3 MD trajectories was done using the *gmx cluster* routine with Gromos clustering method and a distance cutoff of 0.25 nm; these structures were used as an input for protein–protein docking procedure.

The VSD-II/Hm-3 protein–protein docking runs were done using the ZDOCK software (Chen et al., 2003). The allowed contact surfaces in the VSD-II/Hm-3 complex were restricted using standard ZDOCK “block” option. The docking solutions were “filtered” by the PLATINUM software (Pyrkov et al., 2009) (see the Results section for details). The obtained complexes were visually inspected and clustered.

## Results

### Hm-3 Inhibits Gating Pore Currents in R675G Na_V_1.4

**Figure 2:**
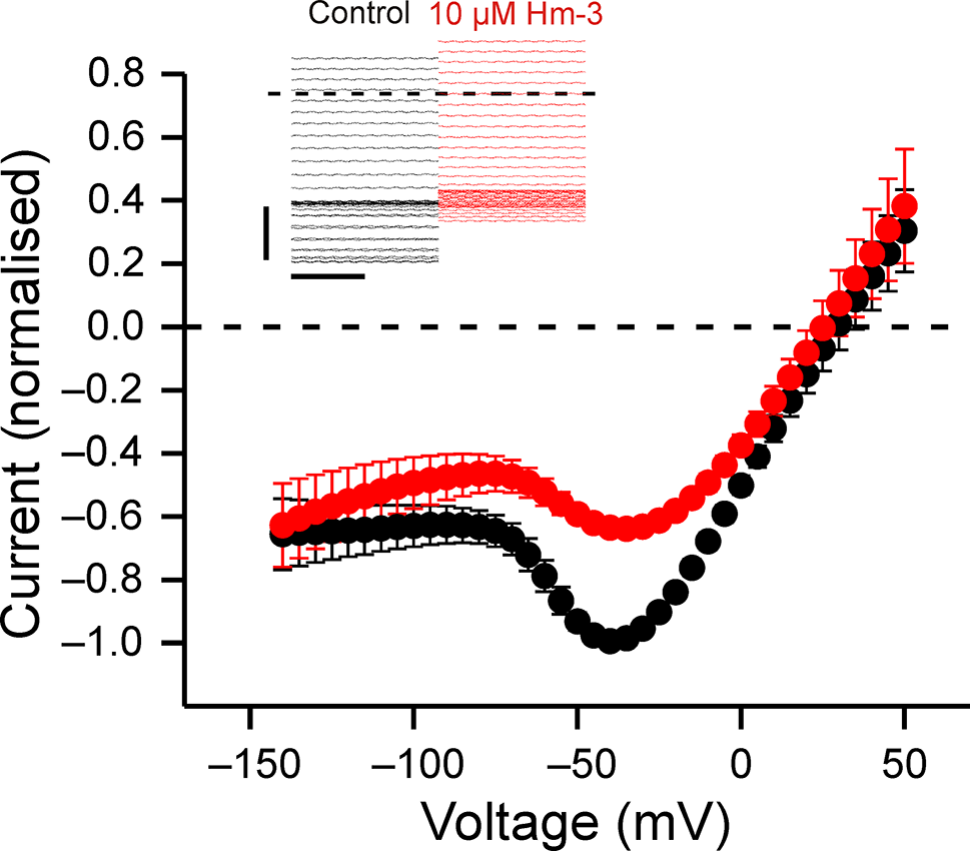
Hm-3 inhibits gating pore currents through Na_V_1.4-R675G. The voltage dependence of gating pore currents in absence (black symbols) and presence (red symbols) of 10-µM Hm-3, n = 4. The currents in each cell were normalized to peak negative current in control condition. The insert shows the last 200 ms of the currents in response to 300-ms pulses to voltages ranging from –140 to 50 mV in 5-mV increments in control condition (black traces) and in the presence of 10-µM Hm-3 (red traces).

Previously, Hm-3 has been shown not to affect gating pore currents through VSD-II with mutated R2 (Männikkö et al., 2018). Further screening of Hm-3 on gating charge mutant channels revealed that gating pore currents through the VSD-II R3 (R675G) mutant were suppressed (**Figure 2**). Analogous to VSD-I R3G, the gating pore currents of VSD-II R3G are increased at depolarized voltages. As in the case of VSD-I R3 (R225G) (Männikkö et al., 2018), the gating pore current was mainly inhibited upon early activating depolarization, with maximal inhibition observed at −60 to −20 mV, while gating pore currents at more depolarized voltages were little affected (**Figure 2**). Consequently, the mode of inhibition is consistent with the toxin locking VSD-II in the down state.

### Cell-Free Production of Voltage-Sensing Domain II

**Figure 3:**
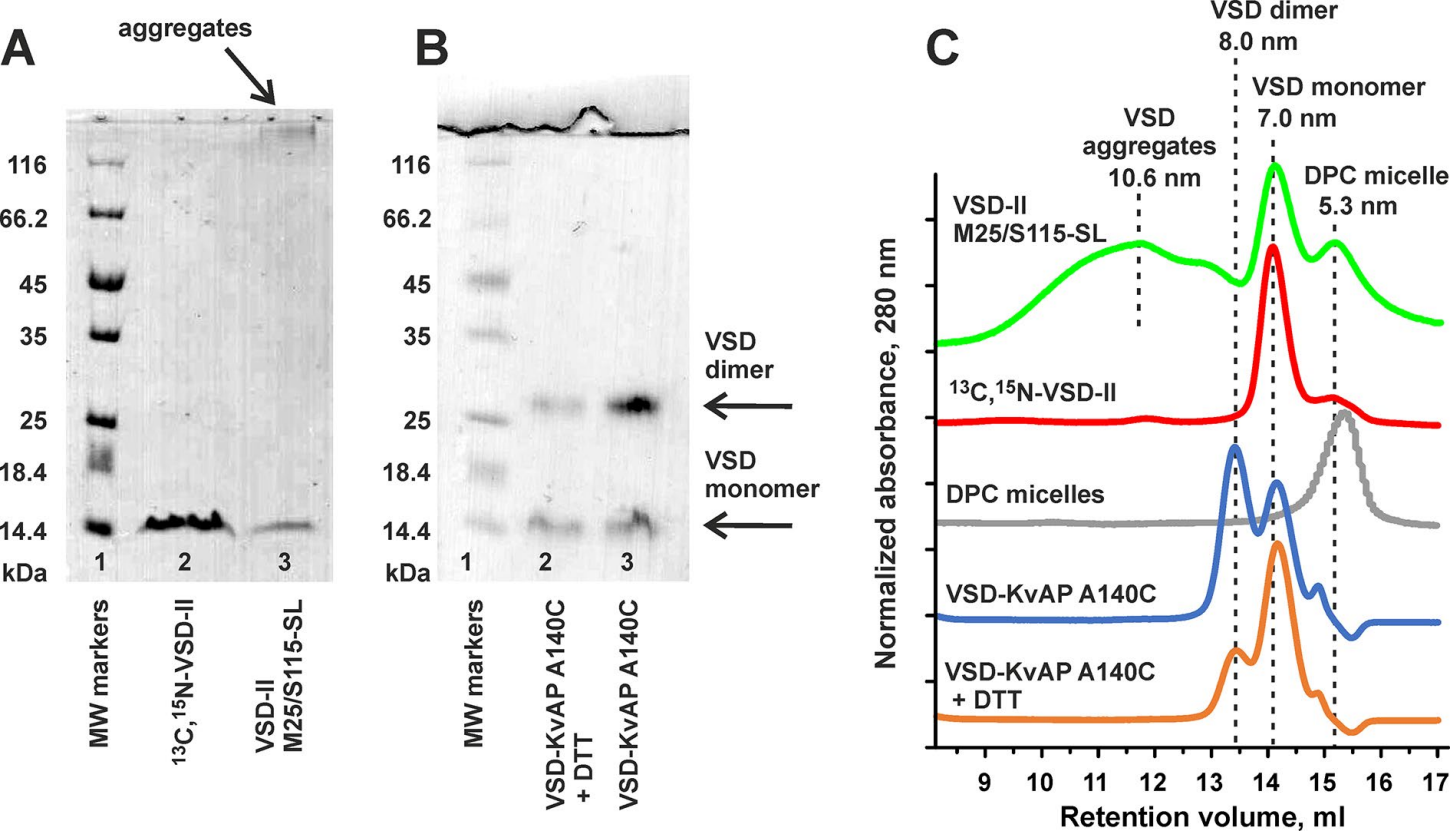
Characterization of cell-free-produced isolated voltage-sensing domain II of Na_V_1.4 channel. **(A)** 12% SDS-PAGE of purified VSD-II. Lane 1, molecular mass markers; lane 2, ^13^C,^15^N-VSD-II after purification by Ni^2+^-chromatography; lane 3, double M25C/S115C VSD-II mutant after labeling with MTSL and Ni^2+^ purification. **(B)** SDS-PAGE of purified A140C mutant of VSD-K_V_AP. Lane 1, molecular mass markers; lanes 2 and 3, protein samples after and before treatment with DTT. Bands corresponding to monomeric and dimeric VSDs are labeled. **(C)** SEC analysis of partially aggregated VSD-II M25/S115-SL, ^13^C,^15^N-VSD-II, empty DPC micelles, VSD-K_V_AP A140C, and VSD-K_V_AP A140C after treatment with DTT.

To explore the molecular mechanism of Hm-3 interaction with VSD-II of Na_V_1.4, we employ the NMR-based approach that has been recently successfully used for the study of Hm-3 complex with VSD-I (Männikkö et al., 2018). The unlabeled, ^15^N-labeled, or ^13^C,^15^N-labeled VSD-II was synthesized in the form of RM precipitate. To transfer the protein into a membrane mimetic, the RM precipitate was solubilized in an excess of DPC and purified by Ni^2+^-chromatography. Sodium dodecyl sulfate polyacrylamide gel electrophoresis (SDS-PAGE) and SEC analysis revealed that VSD-II in complex with DPC micelle forms particles with a characteristic diameter of ∼7.0 nm containing monomeric protein (**Figures 3A, C**). To prove this, we used the sample of the well-characterized VSD of K_V_AP channel. The single A140C mutant of VSD-K_V_AP in DPC environment formed a mixture of monomers (diameter of ∼7.0 nm) and covalent dimers stabilized by an intermolecular disulfide bridge (diameter of ∼8.0 nm). The addition of 1.5-mM DTT led to partial interconversion of the VSD-K_V_AP dimers into monomers (**Figures 3B, C**). Interestingly, the labeling of the VSD-II Cys-mutants by MTSL resulted in the formation of large protein oligomers, observed by SDS-PAGE and SEC (**Figures 3A, C**). Therefore, additional purification by SEC was used for the spin-labeled VSD-II samples.

### Search for Membrane-Mimicking Environment for the Investigation of Voltage-Sensing Domain II/Hm-3 Complex

NMR studies of IMPs require careful selection of membrane-mimicking media for protein solubilization. The media of choice should preserve the protein structure and functionality and provide sufficient quality of the NMR spectra (Shenkarev et al., 2010). Spider GMTs *per se* have affinity to lipid membranes, and lipid molecules play a crucial role in the interaction of the GMTs with their ion channel targets (Milescu et al., 2007). Thus, the environment chosen for NMR study should also preserve VSD/GMT interaction.

**Figure 4:**
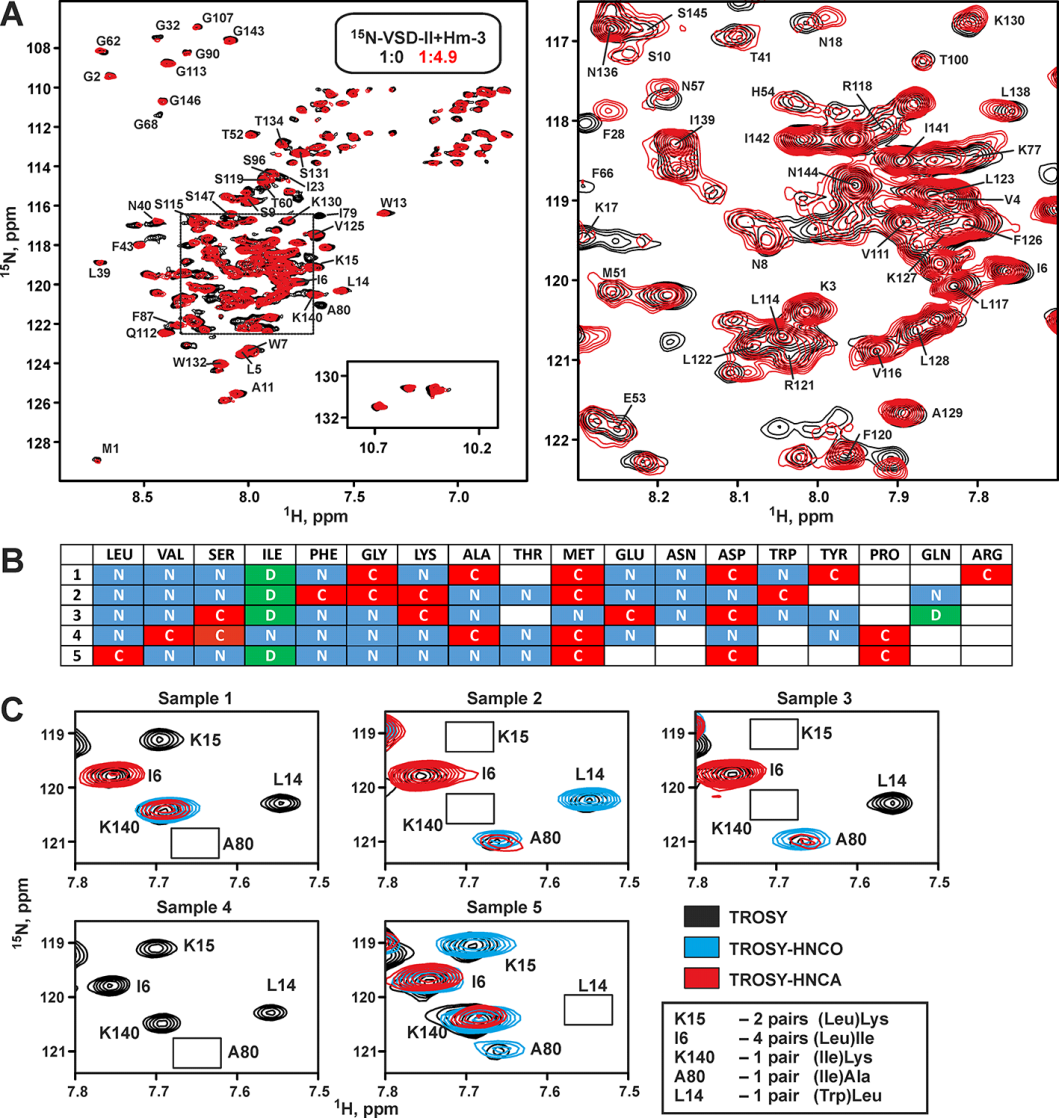
Nuclear magnetic resonance spectra and resonance assignment of voltage-sensing domain II. **(A)** Overlay of ^1^H,^15^N-TROSY spectra of 30-µM ^15^N-labeled VSD-II in DPC/LDAO (11/11 mM, pH 5.5, 45°C, 800 MHz) before (black) and after (red) addition of 150-µM unlabeled Hm-3. Final concentrations: 23-µM VSD-II, 114-µM Hm-3, and detergent to Hm-3 molar ratio of 190:1. The insert on the left panel shows ^1^H^15^N^ε1^ signals of Trp side chains (not assigned). The dashed frame highlights the region expanded in the right panel. The residue numbering scheme corresponds to the expressed VSD-II construct with the *N*-terminal Met1. The observed ^1^H-^15^N cross peak of the Met1 residue indicates the presence of unprocessed *N*-terminal formyl, which is typical for proteins synthesized in CF systems based on bacterial extracts. **(B)** Labeling pattern for five VSD-II samples (rows in the table) used for backbone resonance assignment. Histidine was unlabeled, and cysteine was not used for the CF synthesis. **(C)** An example of ^1^H-^15^N cross peaks assignment. The overlays of 2D TROSY, TROSY-HNCO, and TROSY-HNCA spectra are shown. Absent peaks are marked by rectangles. The degree of ambiguity in the assignment for each of the cross peaks is shown in the insert. The remaining ambiguities were resolved by 3D HNCA and HN(CO)CA spectra measured for uniformly ^13^C,^15^N-labeled VSD-II.

Previously, we have performed screening of membrane-mimicking media for the NMR study of VSD-II (Paramonov et al., 2017). It was found that the protein tends to aggregate in environments containing phospholipid bilayers (e.g., bicelles or nanodiscs). The micelles of anionic lysolipid LPPG provided the best quality of NMR spectra, thus allowing full characterization of the secondary structure, backbone dynamics, and topology of the VSD-II/micelle interaction (Paramonov et al., 2017). However, all attempts to study the VSD-II/Hm-3 complex in this milieu failed. Positively charged (+4) toxin molecule strongly interacted with the anionic lipids, and the Hm-3 NMR signals became significantly broadened (data not shown). Therefore, for the structural study of VSD-II/Hm-3 complex, we have chosen DPC/LDAO micelles. This mixture of zwitterionic detergents has been previously used successfully for the investigation of VSD-I/Hm-3 (Männikkö et al., 2018) and VSD-KvAP/VsTx1 (Shenkarev et al., unpublished) complexes. The observed changes in ^15^N-TROSY spectrum of VSD-II upon Hm-3 addition confirmed that VSD/toxin interaction is preserved in this environment (**Figure 4A**). Interestingly, the stability of VSD-II sample in DPC/LDAO (1:1) micelles was significantly higher than stability of VSD-I in the same experimental conditions (half-life at 45°C of >4 days and ∼24 h, respectively).

Due to a high price of perdeuterated LDAO, the *d* *_38_*-DPC micelles were used for the EPR experiments requiring fully deuterated background (see later discussion). Despite the lower quality of VSD-II NMR spectra in DPC, the pattern of signals and spectral changes observed upon Hm-3 addition were similar to those in DPC/LDAO (**Figure 4A**. and **S1**). Thus, VSD-II has similar spatial structure and ligand binding properties in these environments. The lower quality of spectra in DPC micelles was associated with the higher degree of signal broadening. This reveals differences in the VDS dynamics. Most probably the μs–ms timescale conformational fluctuations in the VSD-II molecule have higher amplitude and/or go much slower in the DPC micelles.

### Combinatorial Selective Labeling and Backbone Assignment of VSD-II in DPC/LDAO Micelles

The VSD-II spectra in DPC/LDAO micelles still demonstrated many broadened signals (**Figure 4A**). This led to a substantial decrease in the sensitivity of NMR experiments and significantly complicated the backbone resonance assignment using classic triple-resonance method, which requires measurement of several 3D spectra for a uniformly ^13^C,^15^N-labeled protein. To speed up the assignment process, combinatorial selective labeling (CSL) was used (Löhr et al., 2012). In this method, the signal of a particular protein residue is identified in a 2D ^1^H,^15^N-correlation spectrum (e.g., TROSY) using the 2D versions of the most sensitive 3D experiments (e.g., HNCO or HNCA). To do that, the corresponding residue of the protein must be ^15^N-labeled, while the previous residue must incorporate ^13^C-label at the C’ and/or C^α^ positions. Labeling of AA residues in a combinatorial way in several protein samples can provide unambiguous backbone assignment.

To use the CSL method, a scheme of isotope incorporation is required, e.g., one needs to specify the number of labeled samples and the labeling pattern for each residue type in each of the samples. For calculation of the optimal CSL scheme, we used the recently developed CombLabel program (https://github.com/mmjmike/CombLabel; Myshkin et al., 2019). This program takes into account the available AA stock, minimizes the number of required selectively labeled samples and their price, and maximizes assignment information. In our stock, we had unlabeled, ^15^N-, ^13^С’-, and ^13^C,^15^N-labeled AAs (further abbreviated as “X,” “N,” “C,” and “D” labeling types), but “C” or “D” labeling were not available for all AAs. The calculated CSL scheme for VSD-II assignment (**Figure 4B**) contained five samples.

**Figure 5:**
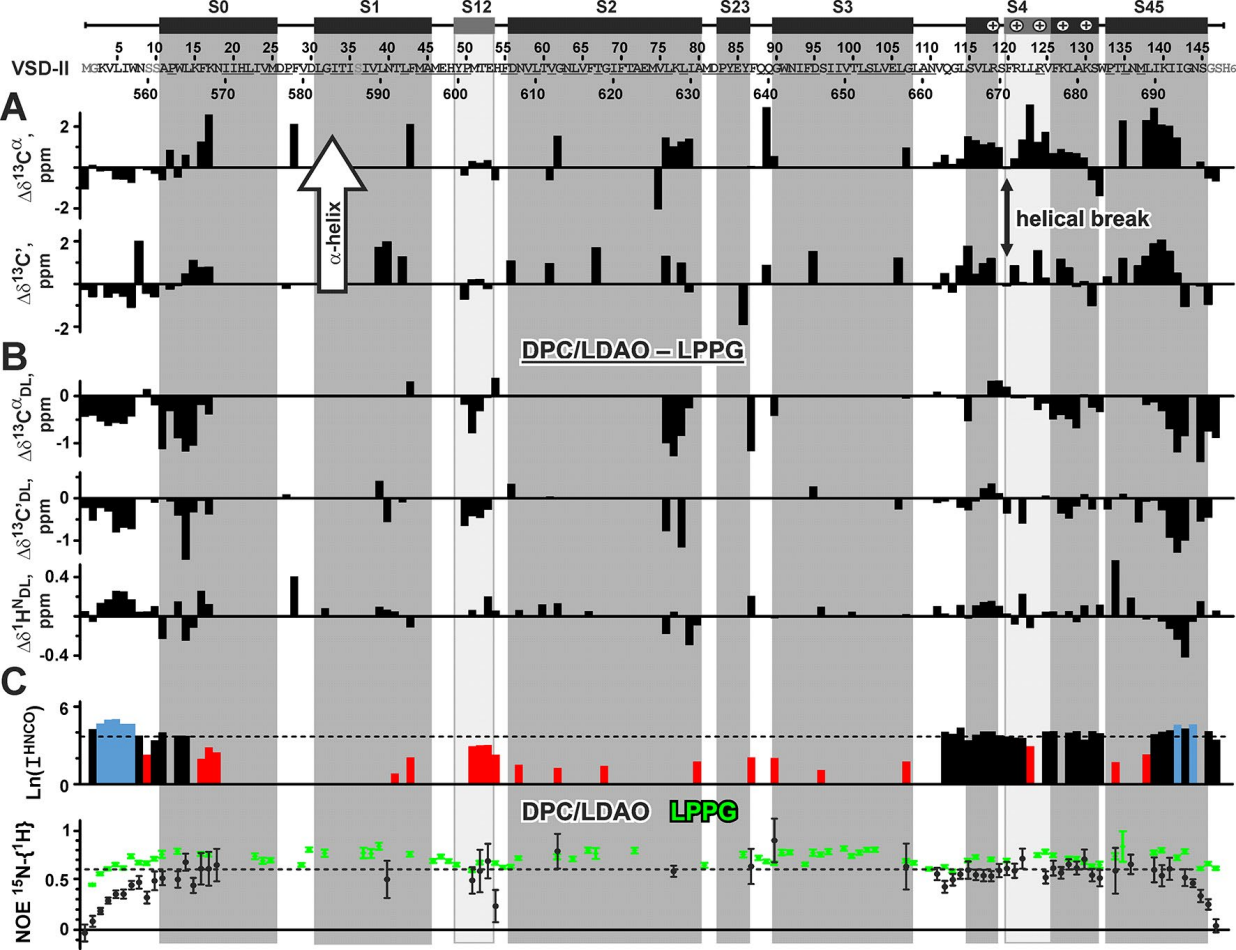
Nuclear magnetic resonance data define the secondary structure and backbone dynamics of voltage-sensing domain II in n-dodecylphosphocholine/n-dodecyl-N,N-dimethylamine-N-oxide micelles. Unassigned residues are underlined. Artificially introduced and mutated residues are in gray. The secondary structure in the published cryo-EM structure of Na_V_1.4 channel (Pan et al., 2018) is shown by bars and vertical shading. The distorted S12 helix and a fragment of 3_10_-helix in the S4 segment are shown in light gray. The conserved Arg residues that are responsible for voltage gating and two Lys residues are marked by “+”. **(A)** Positive values of the Δδ^13^C^α^ and Δδ^13^C’ secondary chemical shifts (the direction is shown by arrow) indicate backbone helical conformation. **(B)** Difference in the VSD ^13^C^α^, ^13^C’, and ^1^H^N^ chemical shifts between DPC/LDAO and LPPG micelles. Positive and negative values of Δδ^13^C^α^_DL_ and Δδ^13^C’_DL_ indicate an increase and decrease of helicity, respectively, upon protein transfer from LPPG to DPC/LDAO. **(C)** Intensity (log values) of peaks in the 3D HNCO spectrum. The level corresponding to the average intensity is shown by a dashed line. Intensities larger than twice the average (blue) reveal residues with high conformational mobility in the ps–ns time scale. Intensities smaller than half the average (red) reveal residues either belonging to the less mobile TM helices or subjected to conformational exchange in the µs–ms time scale. Residues displaying ^15^N-{^1^H} NOE <0.6 are subjected to high-amplitude motions in the ps–ns time scale. For comparison, ^15^N-{^1^H} NOE values for VSD-II in LPPG micelles are shown in green.

Selectively labeled VSD-II variants were produced by CF synthesis using mixtures of labeled and unlabeled AAs. The information from 2D TROSY, TROSY-HNCO, and TROSY-HNCA spectra measured for selectively labeled VSD (**Figure 4C**) together with the data from conventional 3D spectra (HNCO, HNCA, HNCOCA, CBCACONH, and NOESY-^15^N-HSQC) measured for the uniformly ^13^C,^15^N-labeled VSD-II provided a straightforward assignment of 51% backbone amide groups and 44% C’, 45% C^α^, and 23% C^β^ nuclei. The assignment fully covers the N- and C-terminal domain segments including the S4 TM helix, some fragments of the S1, S2, and S3 TM helices, and cytoplasmic and extracellular loops (**Figure 5**).

### Nuclear Magnetic Resonance Data Define the Secondary Structure and Backbone Dynamics of Voltage-Sensing Domain II in n-Dodecylphosphocholine/n-Dodecyl-N,N-Dimethylamine-N-Oxide Micelles

Chemical shifts of the ^13^C^α^ and ^13^C’ nuclei were used to characterize the secondary structure of VSD-II in DPC/LDAO micelles (**Figure 5A**). The data confirmed the formation of α-helices in the segments S0, S4, and S45. At the same time, the S4 helix was found to be subdivided in two parts by a break at the residue F120(671) (residue numbers in the full-length Na_V_1.4 channel are given in parentheses). The individual positive values of Δδ^13^C^α^ and Δδ^13^C’ also pointed out the presence of α-helical conformation in the segments S1, S2, and S3. On the other hand, the chemical shift data indicated the absence of helical conformation in the interhelical loops and confirmed the distortion of the S12 helical element. The presence of helical break at F120(671) and distortion of S12 correspond nicely to the recently published cryo-EM structure of the full-length Na_V_1.4 channel (Pan et al., 2018). In that structure, the S119(670) residue divides the S4 helix into two parts (α-helical and 3_10_-helical, **Figure 5A**).

To compare the secondary structure of VSD-II in zwitterionic DPC/LDAO micelles with its secondary structure previously determined in the environment of anionic lysolipid LPPG (Paramonov et al., 2017), we calculated differences in the chemical shifts of ^13^C^α^ and ^13^C’ nuclei (**Figure 5B**). The obtained Δδ^13^C^α^_DL_ and Δδ^13^C’_DL_ values indicated that the VSD-II molecule has lower helicity in the DPC/LDAO micelles. Only one region of the S4 helix around F120(671) demonstrated slight increase in the helical content as compared with LPPG micelles. To compare the overall spatial structure of VSD-II in different environments, we calculated differences in the chemical shifts of backbone amide protons (**Figure 5B**). The ^1^H^N^ chemical shifts are very sensitive to the long-range contacts in the protein structure, e.g., to hydrogen bonding, helix bending, and spatial proximity of charged and aromatic groups. The calculated Δδ^1^H^N^_LD_ values were relatively small (∼0.12 ppm on average). The largest δ^1^H^N^ changes (0.4–0.5 ppm) were observed at the peripheral parts of the protein: in the loop between S0 and S1 helices [residue F28(579)] and in the C-terminal S45 helix [residues T134(685) and G143(694)]. Thus, the TM region of VSD-II has similar spatial organization in the DPC/LDAO and LPPG micelles.

To investigate the backbone mobility of VSD-II, we analyzed the intensities of cross peaks in the 3D HNCO spectrum and steady-state ^15^N-{^1^H} NOE values (**Figure 5C**). The significant spread of HNCO intensities indicated the presence of intramolecular motions at different time scales. Intense cross peaks define the protein regions participating in the high-amplitude ps–ns time scale motions. According to these data, the N- and C-terminal regions and the S4 helix of VSD-II are significantly more mobile at the ps–ns time scale as compared with the S1, S2, and S3 helices. The relatively low ^15^N-{^1^H}-NOE values (from 0.5 to 0.7, **Figure 5C**) confirmed this observation. Moreover, the comparison with NOE values previously observed in the LPPG micelles revealed that the VSD-II backbone has increased ps–ns mobility in the DPC/LDAO environment. The most drastic changes were observed in the *N*- and *C*-terminal regions. These changes are consistent with the tendency of LPPG to induce artificial stabilization of the solvent-exposed or peripheral regions of IMPs (Shenkarev et al., unpublished). We propose that the observed increase in ps–ns mobility is the main reason of the decrease in VSD helicity in the DPC/LDAO environment as compared with LPPG. On the other hand, the weak HNCO intensities observed in the S1, S2, and S3 helices revealed the presence of high-amplitude μs–ms time scale conformational fluctuations in the TM part of VSD-II (**Figure 5C**).

### Double Electron–Electron Resonance Revealed the Quasi-Native Fold of Voltage-Sensing Domain II in the Micellar Environment

**Figure 6:**
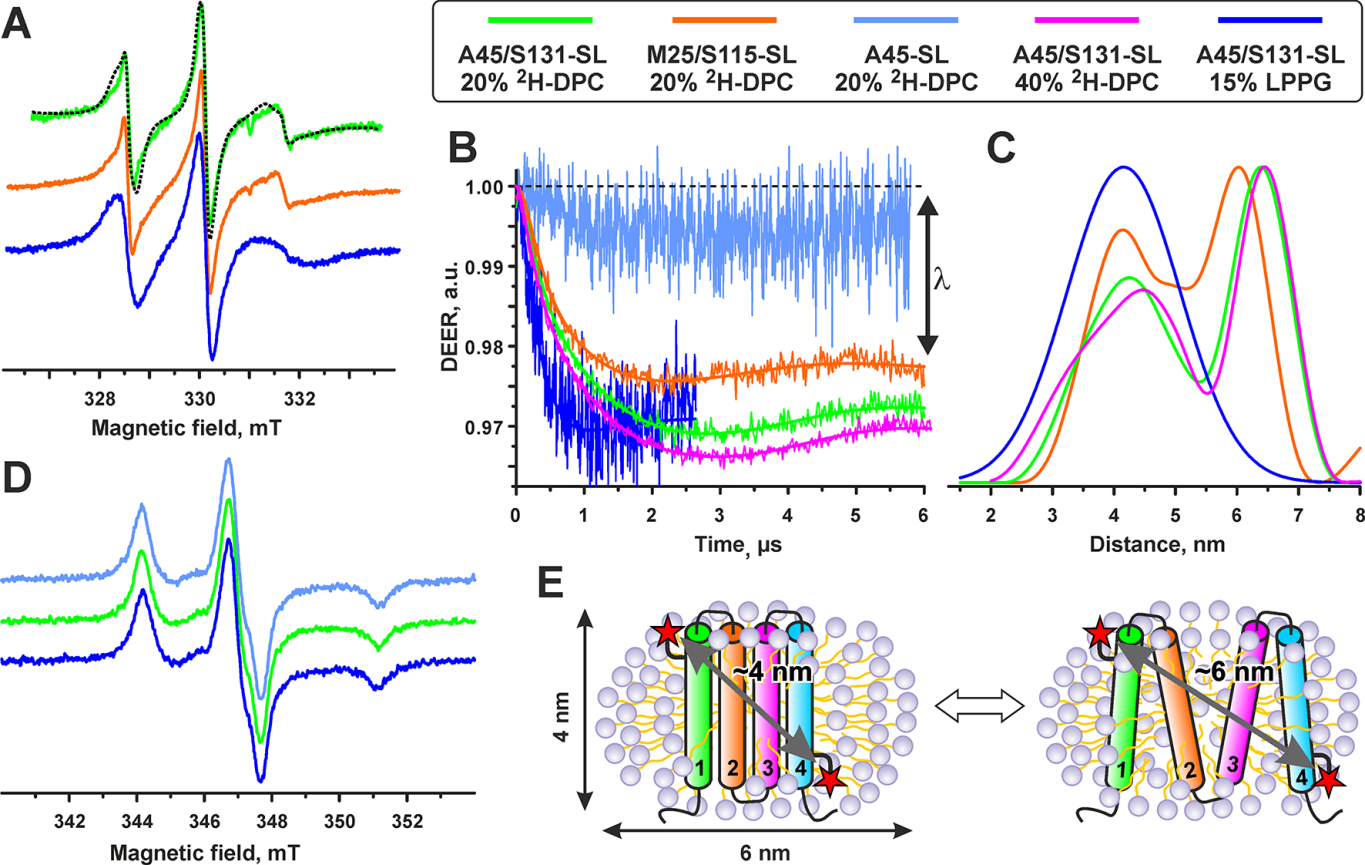
Electron paramagnetic resonance data reveal partial unfolding of spin-labeled voltage-sensing domain II variants in DPC micelles. **(A)** X-band CW EPR spectra of the A45/S131-SL and M25/S115-SL VSD-II variants in 20% *d* *_38_*-DPC and 15% LPPG at 300 K. The dashed line shows a simulated spectrum obtained assuming a mixture of the free spin-label (8%) with the isotropic motion with τ_c_ = 0.4 ns and of the spin-label attached to the protein (92%) having anisotropic motion with τ_c_ = 1 ns. **(B)** Distance measurements in single- and double-spin labeled VSD-II variants in different membrane-mimicking media (see legend above the figure). Background-corrected four-pulse Q-band DEER traces are shown with normalized intensity. Solid lines show the best fits obtained using DeerAnalysis2013. **(C)**. Interspin distance distributions obtained from the analysis of the DEER traces. In all cases, the regularization parameter L is set to 1,000. **(D)** X-band CW EPR spectra of the A45/S131-SL and A45-SL variants of VSD-II at 140 K. The spectra of the M25/S115-SL variant are identical and are not shown in the figure. **(E)** Cartoon depicting folded and partially unfolded VSD-II in complex with a DPC micelle.

To investigate the tertiary structure of VSD-II in detergent micelles, we applied pulsed and CW EPR spectroscopy. To minimize the distortions in the TM part of VSD-II by the introduced spin-labels and provide spin–spin distances in the range accessible to the DEER measurements (>2 and <8 nm), we placed the spin-labels on the cytoplasmic and extracellular borders of the TM helices. Two double-Cys mutant variants of VSD-II were obtained by CF synthesis (A45C/S131C and M25C/S115C) and were labeled by the nitroxide spin-label MTSL. First variant A45/S131-SL contained spin-labels at the C-termini of the S1 and S4 helices, while the second variant M25/S115-SL contained the labels at the N-termini of these helices. Thus, in both cases, the theoretical spin–spin distances in the folded domain should have been approximately equal to the membrane (micelle) width (∼4.0 nm, **Figure 6E**, the length of Cys-MTSL group, ∼1 nm was taken into account). In case of the VSD unfolding, the spin–spin distances should be much longer. Additional single-Cys mutant and corresponding single-labeled A45C-SL variant of VSD-II was obtained for control EPR measurements.

The attachment of the labels was verified by the analysis of room-temperature CW EPR spectra (**Figure 6A**): the spin-label concentrations measured by EPR agreed well with the protein concentration measured by UV-Vis spectroscopy. The main contribution (>90%) to the EPR spectra was assigned to spin-labels having relatively slow (rotational correlation time τ_c_ ∼1 ns in DPC micelles) and anisotropic motion. This indicates a partial ordering of the spin-label within its local environment occurring due to the covalent attachment to the VSD. The τ_c_ values were found independent on the position of spin-labels attachment but dependent on the detergent composition (τ_c_ values in LPPG were ∼1.4 ns).

The interspin distances in the double spin-labeled VSD-II variants were measured by Q-band DEER spectroscopy at 50 K. Initial experiments in 3–5% DPC (protein/detergent molar ratio >500) revealed a relatively short T_2_ relaxation time of the spin-labels (the phase memory time T_m_ of 1.2–1.5 µs). With these T_2_ values, the measurements of interspin distances beyond 4 nm were impossible. An increase of detergent concentration to ∼20% and use of fully deuterated environment (*d* *_38_*-DPC and D_2_O) significantly lengthened the T_2_ time (T_m_ of 4–5 µs) and enabled us to record DEER time-traces up to 6 µs (**Figure 6B**).

The DEER time-traces of both double-labeled VSD-II variants demonstrated pronounced dipolar oscillations, but we did not observe any dipolar oscillations for the single-labeled A45-SL VSD-II variant (**Figure 6B**). This indicates the absence of close intermolecular contacts between two different VSD molecules. An increase of DPC concentration to 40% in the A45/S131-SL sample did not change the overall shape of the DEER signal but slightly increased the modulation depth λ (from 2.8 to 3.0%). This confirmed the absence of VSD dimerization or formation of higher order aggregates in the samples. Thus, all observed distances for double-labeled samples should be attributed solely to the spin–spin interactions within individual VSD-II molecules. The value of modulation depth λ depends on the fraction of spin pairs contributing to the dipolar modulation. The observed λ values (∼3 and ∼2.3% for A45/S131-SL and M25/S115-SL, respectively) are very close to the expected maximum (3%) for the used DEER experimental setup (see Experimental section). Thus, almost all spins in the samples reside in pairs with average distances less than 7 nm.

The interspin distance distributions in both double-labeled VSD-II variants clearly displayed two major peaks (**Figure 6C**). The mean values <R_DEER_> of the first peak were the same for both A45/S131-SL and M25/S115-SL variants (4.2 ± 0.7/0.5 nm, mean ± s.d.). This value is in good agreement with the distances expected in the four-helix bundle of folded VSD-II (**Figure 6E**). The second peak with <R_DEER_> of 6.4 ± 0.5 nm and 6.1 ± 0.5 nm for A45/S131-SL and M25/S115-SL, respectively, can be assigned to a partially unfolded VSD. The observed distances correspond nicely to the dimensions of the DPC micelle (**Figure 6E**), which represents a prolate ellipsoid with dimensions along the main axes of about 4 and 6 nm (Lipfert et al., 2007). Thus, the unfolded VSD-II is probably associated with one DPC micelle (the length of extended α-helix with >90 residues should be significantly larger, ∼14 nm). The integrals over obtained distance distributions (**Figure 6C**) revealed that the folded and unfolded VSD-II conformations are almost equally populated.

In contrast to *d* *_38_*-DPC, the T_m_ value of A45/S131-SL VSD-II in 15% LPPG was relatively short (1.7 µs), probably due to the influence of protonated environment. Therefore, the DEER observation time-window was limited by 2.5 ms (**Figure 6B**). The distance distribution presented in **Figure 6C** shows one peak with <R_DEER_> = 4.2 ± 0.9 nm. Note that only the mean distance can be analyzed with such a short DEER time-trace, whereas the width and the shape of the distribution are not reliable. The observed value of the modulation depth λ ∼3% indicates that almost all spins in the sample are in pairs with average distances less than 5 nm.

For additional validation, we performed CW EPR measurements at 140 K (**Figure 6D**). The spectra of double-labeled variants in DPC and LPPG micelles were identical to the spectrum of the single-labeled A45-SL VSD-II, indicating the absence of interspin distances shorter than 2 nm (Banham et al., 2008). Thus, all spin–spin distances presented in the system fall into the ranges of 2–7 nm (DPC) and 2–5 nm (LPPG) and are detectable in our DEER experiments.

The obtained data revealed that the spin-labeled VSD-II molecule in LPPG environment represents a folded four-helix bundle, whereas in DPC micelles, the domain undergoes partial unfolding, with relative populations of the folded and unfolded states being close to 1:1. At the same time, the analysis of NMR spectra of non-spin-labeled VSD-II in DPC and DPC/LDAO environments revealed only one set of backbone resonances indicating the presence of a single VSD-II structural state. The close correspondence of the chemical shifts (**Figure 5B**) indicated that the VSD-II conformation in DPC/LDAO is similar to the folded domain in LPPG. Thus, the observed unfolding is the result of the attachment of MTSL labels that destabilize the VSD-II spatial structure in the DPC environment. This destabilization is also possibly responsible for the partial oligomerization of the spin-labeled domain (**Figure 3C**). To confirm this hypothesis, we produced an ^15^N-labeled M25C/S115C VSD-II variant, labeled it with MTSL, and reduced the spin-labels by ascorbic acid. The ^15^N-HSQC spectrum of the double ^15^N/MTSL-labeled VSD-II in DPC micelles demonstrated significant line broadening and disappearance of some resonances as compared with the ^15^N-labeled sample (Figure S2). Thus, the attachment of MTSL groups leads to formation of several VSD-II structural states in the DPC solution. Previously, a similar effect associated with partial unfolding was observed for the MTSL-labeled variants of isolated VSD-K_V_AP in DPC but not in LPPG micelles (Paramonov et al., unpublished).

### Nuclear Magnetic Resonance Titrations Reveal Voltage-Sensing Domain II/Hm-3 Interaction Interfaces

**Figure 7:**
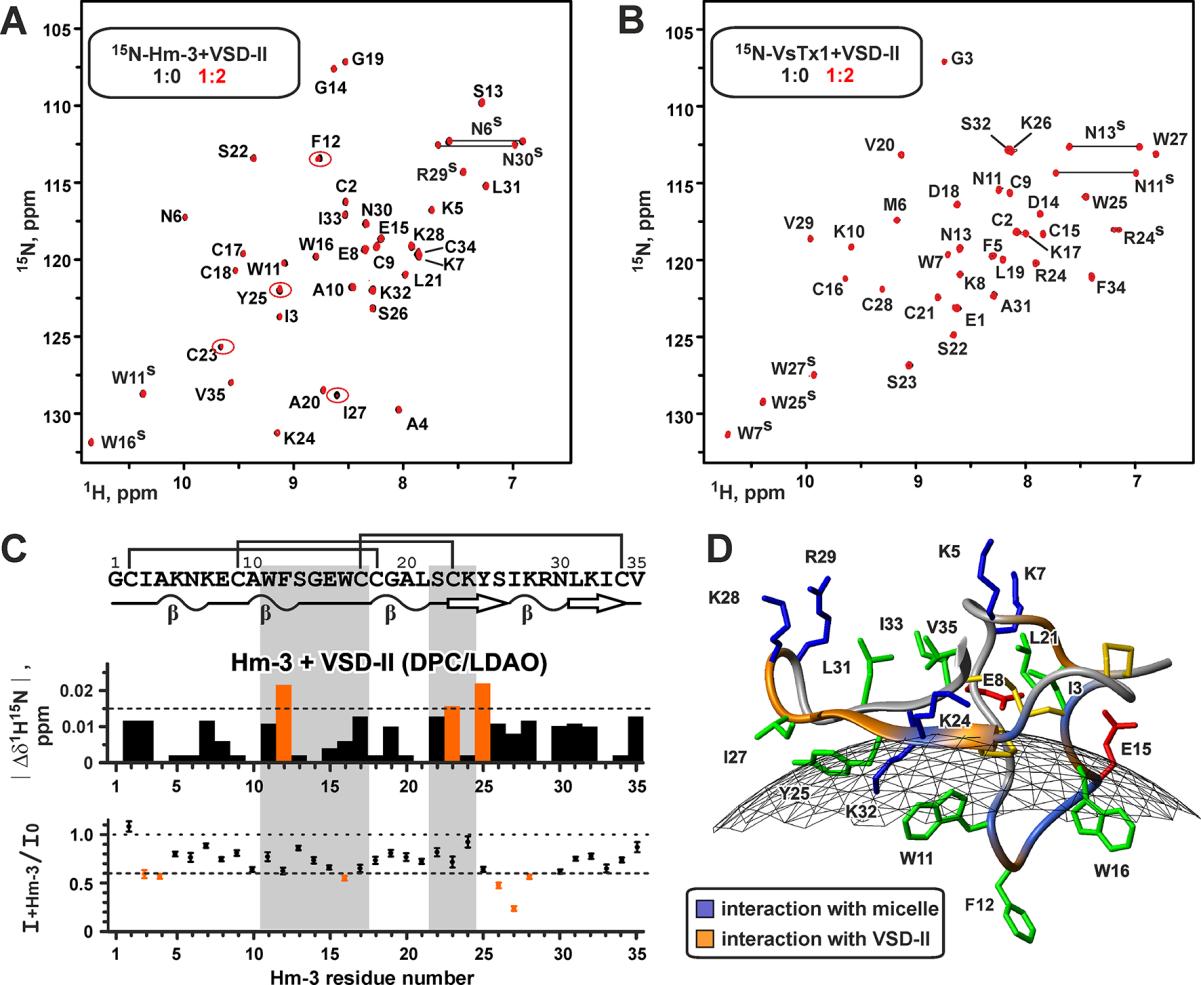
Nuclear magnetic resonance data define the interface of the Hm-3 interaction with voltage-sensing domain II. **(A)** Overlay of ^1^H,^15^N-HSQC spectra of 30-µM ^15^N-labeled Hm-3 in 57/57-mM DPC/LDAO (pH 5.5, 45°C, 800 MHz) before (black) and after (red) addition of unlabeled VSD-II. Final concentrations: 20-µM Hm-3, 40-µM VSD-II, and detergent to Hm-3 molar ratio of 5,700:1. The signals demonstrating the biggest changes in the intensity or chemical shifts are marked by ellipses. **(B)** Overlay of ^1^H,^15^N-HSQC spectra of 30-µM ^15^N-labeled VsTx1 in 45/45-mM DPC/LDAO (pH 5.5, 45°C, 800 MHz) before (black) and after (red) addition of unlabeled VSD-II. Final concentrations: 20-µM VsTx1, 40-µM VSD-II, and detergent to VsTx1 molar ratio of 4,500:1. **(C)** Relative changes of the ^1^H-^15^N chemical shifts and intensities of the Hm-3 resonances upon addition of VSD-II (VSD-II/Hm-3 molar ratio of 2:1). Dilution of the Hm-3 sample was accounted for during calculation of the intensity ratio. The threshold levels of 0.015 ppm and 0.6 are shown by dotted lines. Hm-3 residues involved in the interaction with DPC/LDAO micelle are highlighted in gray. **(D)** The interfaces of the Hm-3 interaction with the micelle (blue) and VSD-II (orange) are mapped on the Hm-3 structure. Gray mesh shows approximate micelle surface with a radius of ∼24 Å.

Previously, we have shown that Hm-3 has affinity to DPC/LDAO mixed micelles and binds to the micelle surface by the loop region containing aromatic residues W11, F12, and W16 (**Figure 7D**) (Männikkö et al., 2018). To map the interaction interfaces in the VSD-II/Hm-3 complex, we titrated the ^15^N-labeled toxin with unlabeled VSD-II. To prevent the change of Hm-3 partitioning between water solution and micelles, detergent concentration was kept constant during the experiments. The titration revealed changes in chemical shifts and intensities of the Hm-3 ^1^H-^15^N signals (**Figures 7A, C**). The largest changes were observed for the *N*-terminal strand and the tip of the β-hairpin (residues C23-K28) and for the F12 residue at the membrane-binding face (**Figure 7D**). This β-hairpin region accommodates three positively charged residues (K24, K28, and R29), which may interact with VSD-II. The absence of significant chemical shift changes for other residues from the membrane-binding face of the toxin (residues A10-C17) indicates that the topology of the Hm-3/micelle interaction is not significantly altered upon toxin binding to VSD-II.

To confirm that the observed changes are due to specific toxin–VSD interactions, we used another spider toxin VsTx1 from *Grammostola rosea*. VsTx1 also belongs to the GMT family but targets other K_V_ and Na_V_ channels, having very week activity against Na_V_1.4 (Redaelli et al., 2010). Detergent titration experiments revealed that VsTx1 also binds to DPC/LDAO micelles (**Figure S3**). Titration of micelle-solubilized ^15^N-labeled VsTx1 with unlabeled VSD-II did not reveal any changes in the intensity and position of the ^1^H-^15^N signals, indicating the absence of detectable toxin–VSD interactions (**Figure 7B**). It should be noted that VsTx1 is able to interact with isolated VSD of the K_V_AP channel in the same experimental conditions (Shenkarev et al., unpublished).

**Figure 8:**
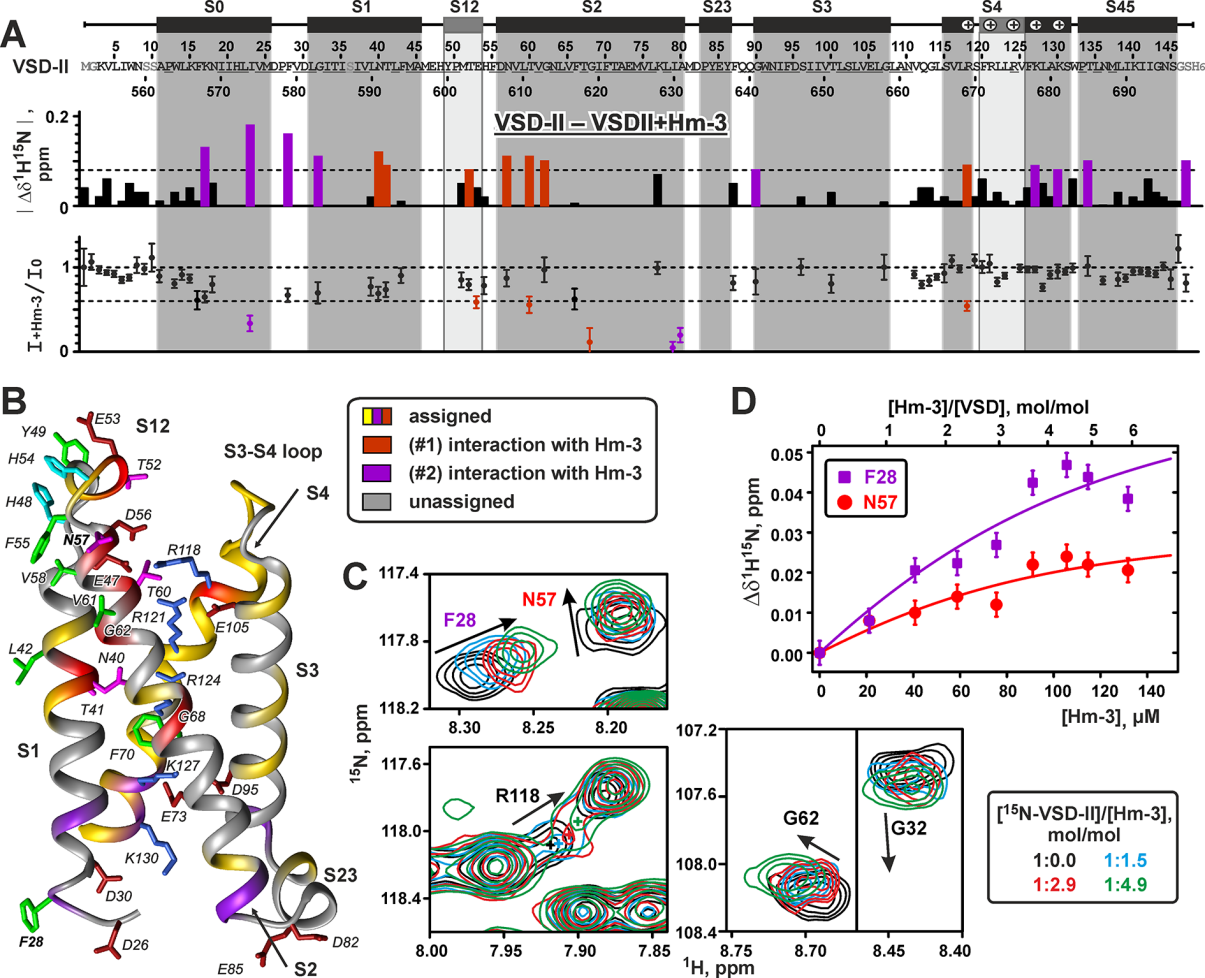
Nuclear magnetic resonance data define the interfaces of voltage-sensing domain II interaction with Hm-3. **(A)** Relative changes in the ^1^H-^15^N chemical shift and in the intensity of VSD-II resonances upon addition of Hm-3 (pH 5.5, 45°C, 800 MHz). Final concentrations: 23-µM VSD-II, 114-µM Hm-3, and 11/11-mM DPC/LDAO. Dilution of the VSD-II sample was accounted for during calculation of the intensity ratio. The threshold levels of 0.08 ppm and 0.6 are shown by dotted lines. **(B)** The interface of VSD-II interaction with Hm-3 is mapped on the structure of VSD-II. The residues forming the extracellular and cytoplasmic Hm-3 binding sites are shown in red and magenta, respectively. The side chains forming the extracellular interaction interface are annotated. The conserved Arg/Lys residues of the S4 helix, negatively charged Asp/Glu residues, and conserved F70(621) residue are also shown. The residue numbering scheme corresponds to the expressed VSD-II construct with the *N*-terminal Met1. **(C)** Fragment of the ^1^H,^15^N-TROSY spectra of ^15^N-labeled VSD-II at different Hm-3 concentrations. **(D)** The binding curves of Hm-3 to VSD-II in DPC/LDAO micelles measured for F28(579) and N57(608) residues (cytoplasmic and extracellular binding sites, respectively).

Titration of ^15^N-labeled VSD-II with unlabeled toxin revealed changes of ^1^H-^15^N signals in several regions of the domain (**Figures 8A, B**). These changes demonstrated two putative Hm-3 binding sites on the VSD surface. The first site is located on the extracellular part of the domain and involves residues of the S1 and S2 helices and S1-S2 loop (**Figures 8A, B**, red). This region of the domain accommodates three negatively charged residues [E47(598), E53(604), and D56(607)], which may form ionic bridges with the Lys and Arg residues of the toxin. In the VSD-II structure, the side chains of E47(598) and D56(607) stabilize the first gating charge of the S4 helix [R1; R118(669)] in the position close to the extracellular surface of the membrane. Indeed, we observed significant changes in the chemical shift and intensity of the R118 signal (**Figure 8C**), thus indicating that Hm-3 binding perturbs these intra-domain electrostatic interactions.

Another putative Hm-3 interaction site was located on the intracellular part of VSD-II and involved residues of the loops between S0 and S1, and S2 and S3 helices (**Figures 8A, B**, magenta). These regions contain two pairs of negatively charged residues [S0-S1: D26(577) and D30(581); S2-S3: D82(633) and E85(636)], which may attract the positively charged toxin molecule. In addition, we observed the changes of NMR signals in the neighboring part of the S4 helix, including the fourth and fifth positively charged residues [K4, K127(678), and K5, K130(681)]. This part of S4 is spatially close to the S1 helix, and K4 and K5 residues participate in electrostatic interactions with the charged groups of the S1, S2, and S3 helices. It should be noted that the cytoplasmic side of the native Na_V_1.4 channel inserted into cellular membrane is inaccessible to the toxin molecules. Therefore, our observation of Hm-3 binding to a cytoplasmic VSD site is an artifact of the used micellar environment, where both extracellular and cytoplasmic sides of the domain are accessible to the toxin. Interestingly, the VSD-I molecule does not contain the pairs of negatively charged residues in the homologous positions of S0-S1, S1-S2, and S2-S3 loops. This explains why, previously, we have not observed Hm-3 interaction with the extracellular S1-S2 loop and intracellular interface of VSD-I (Männikkö et al., 2018).

Hm-3 binding did not influence the NMR signal of R2 [R121(672)] residue, while R3 [R124(675)] remains unassigned. Despite this, we observed changes in NMR signals from residues in the S1 and S2 helices [N40(591), T41(592), and G68(619)] located on the same level as R3 in the Na_V_1.4 crystal structure (Pan et al., 2018) (**Figure 8B**). At present, we cannot define which of the toxin binding interfaces (extracellular or cytoplasmic) is coupled with the changes in the central parts of the S1 and S2 helices. Nevertheless, the observed changes of the VSD-II resonances imply that Hm-3 binding induces conformational perturbations not only at the putative binding sites located in the water/membrane interphase but also deeper in the membrane.

The observed dependence of VSD-II chemical shifts on the toxin/domain molar ratio in the sample (**Figure 8D**) allowed us to estimate the equilibrium dissociation constants (*K* *_V_*) of the VSD-II/Hm-3 complexes. The obtained *K* *_V_* values (∼11 and ∼7 μM for extracellular and cytoplasmic binding sites, respectively) corresponded to the free energies of the complex formation (ΔG*_V_*) of about –7.2 and –7.5 kcal/mol. Thus, the extracellular and cytoplasmic VSD-II binding sites have very similar affinity to the Hm-3 toxin. The obtained *K* *_V_* and ΔG*_V_* values for the VSD-II/Hm-3 complexes can be compared with Hm-3 affinity to the site on the micelle surface (*K* *_M_* ∼90 μM) and free energy of VSD-I/Hm-3 complex formation (ΔG*_V_* ∼–7.6 kcal/mol) determined previously (Männikkö et al., 2018).

### Computational Modeling of the Voltage-Sensing Domain II/Hm-3 Complex

The determined binding interfaces were used to model the VSD-II/Hm-3 complex based upon known spatial structures of Na_V_1.4 channel and the toxin. The intracellular Hm-3 binding site is functionally irrelevant; therefore, we modeled toxin binding to the extracellular VSD-II site only. To model the VSD-II/Hm-3 complex, we used a customized protein–protein ensemble docking procedure subdivided into several steps.

**Figure 9:**
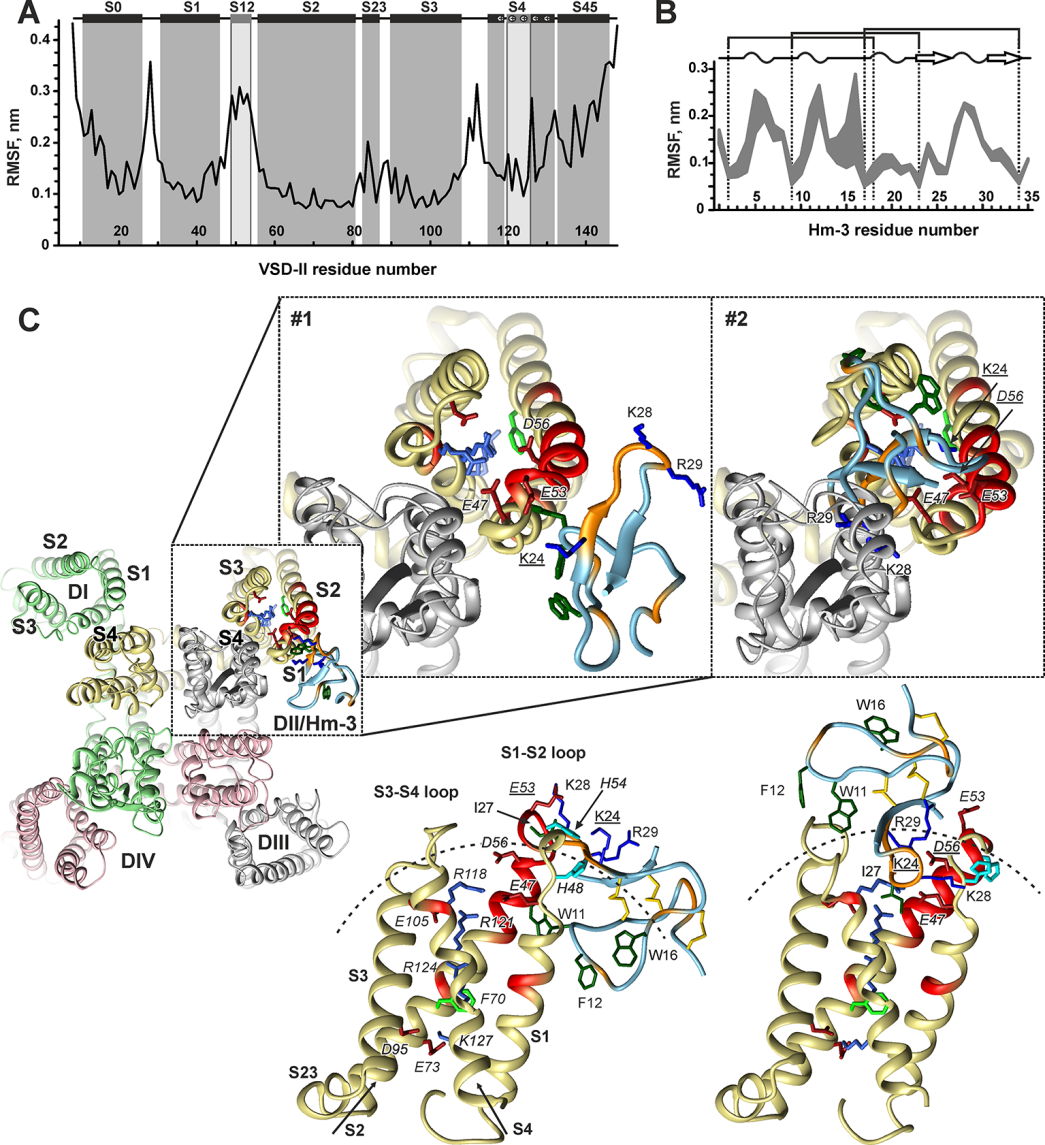
Molecular modeling of Hm-3 in complex with voltage-sensing domain II. **(A)** RMSF of VSD-II calculated over the “stable” part of the MD trajectory. Secondary structure of the Na_V_1.4 cryo-EM structure is shown (Pan et al., 2018). Distorted helices are in light gray. **(B)** RMSF of Hm-3 calculated over the “stable” parts of the MD trajectories (shown is the spread for three independent runs). The toxin secondary structure is shown above; the dotted lines indicate the positions of Cys residues. **(C)** Representative docking solutions of the VSD-II/Hm-3 complex after filtration. The backbone of the fragments, which according to the NMR data form the interaction interfaces, is colored in red (VSD-II) and orange (Hm-3). The charged side chains at the interaction interfaces and in the TM part of VSD-II are shown. The VSD gating charge transfer center (residue F70) and aromatic side chains at the toxin membrane-binding face are also shown. VSD-II residues are in italics type. The residues participating in intermolecular ionic interactions are underlined. Dashed arches in the lower panels show an approximate micelle surface with the radius of ∼24 Å. The upper panels show the position of Hm-3 relative to the full-length α-subunit of Na_V_1.4 (reconstruction based on docking data).

VSD-II (residues 559-699) was extracted from the Na_V_1.4 cryo-EM structure (Pan et al., 2018) (PDB ID 6AGF). This structure captures VSDs in the activated up-state. To account for possible flexibility of VSD-II, we produced an ensemble of conformationally distinct states of the domain using 200-ns MD simulation in a hydrated three-component mixed phospholipid/cholesterol bilayer (POPC/POPE/CHOL = 2:1:1). This lipid composition resembles the membrane composition of cultured dorsal root ganglia neuronal cells (Calderon et al., 1995) and, previously, was successfully used in computational studies of ion channels and ligand–receptor interactions (Lyukmanova et al., 2015; Chugunov et al., 2016). The calculations of root-mean-square fluctuation (RMSF) over the obtained MD trajectory (**Figure 9A**) revealed an increased mobility of the interhelical loops and *N*- and *C*-terminal parts of the domain. Among them, the extracellular S1-S2 and S3-S4 loops, including the S12 helix, demonstrated high amplitude motions. The conformational clustering of the obtained trajectory resulted in the ensemble of four distinct VDS-II conformations, which were used further in protein–protein docking calculations.

Analogously, three MD trajectories of 200 ns each were calculated for Hm-3 in water [starting from three most unlike NMR models, PDB ID 2MQU (Berkut et al., 2015)]. RMSF values in the obtained MD trajectories (**Figure 9B**) revealed an increased mobility in the inter-cysteine loops and in the tip of the β-hairpin. Conformational clustering yielded 10 distinct toxin structures.

Using the obtained domain and toxin structures, 4 × 10 = 40 independent protein–protein docking runs were performed. To exclude possible artifacts due to sparse NMR data, the allowed contact surface of VSD-II in the toxin/domain complex was restricted to residues L39(590)–G68(619) and L101(652)–L122(673). These regions fully covered the extracellular half of the domain, providing almost unrestricted sampling of the toxin binding orientation. The allowed contact surface of Hm-3 was restricted to residues C9–W16 and C23–I33, as identified by changes in the chemical shifts and resonance intensities (**Figure 7C**). For each docking run, 2,000 models of the complex were systematically generated, and 100 top-scoring structures were used for further analysis (4,000 in total).

The docking solutions were “filtered” using our in-house rescoring protocol requiring that: 1) Hm-3 had a significant contact area with the receptor (>250 Å^2^); 2) the number of “good” contacts (H-bonds, ionic bridges, and specific stacking) was three or more; 3) the complementarity of hydrophobic/hydrophilic properties in the complex was >0.4 (Efremov et al., 2007); 4) at least one of the toxin residues K24, K28, and R29 formed an ionic bridge with the VSD; 5) the distance between toxin residue I27 and the VSD was <4 Å (the NMR signal of I27 completely disappeared upon complex formation); and 6) Hm-3 orientation relative to the expected micelle surface is close to the toxin orientation in the Hm-3/DPC/LDAO complex (the W11/F12/W16 hydrophobic cluster is directed toward the membrane, **Figure 7D**). This filtration and visual inspection of the VSD-II/Hm-3 complexes left us with eight solutions, which can be represented by two structural clusters (**Figure 9C**, models #1 and #2).

In the first model (**Figure 9C**, **Table 1**, #1), the toxin interacts peripherally with the S1-S2 loop of VSD-II and contacts directly the “toxin-sensitive” residues on the S1 and S2 helices identified by NMR study. The complex is stabilized by the ionic interaction between the E53(604) of VSD-II and toxin residue K24 (**Table 1**). The side chains of W11, F12, and W16 toxin residues are immersed into the hydrophobic region of the micelle. The mapping of Hm-3 molecule to the full-length Na_V_1.4 channel structure revealed that the toxin backbone does not overlap with the backbone of the channel pore domain (**Figure 9C**, model #1).

**Table 1:** Statistics and interactions in the modeled voltage-sensing domain II/Hm-3 complexes.^a^

	Model #1	Model #2		Model #1	Model #2
ZDock score^b^	1098.7	1077.8	Number of “good” contacts	3.6	4.8
Contact area in the complex (Å^2^)	289	369	Complementarity of hydrophobic/hydrophilic properties	0.63	0.42
VSD-II residue S1-S2 loop	Hm-3 residue		VSD-II residue S3-S4 loop	Hm-3 residue	
L39(590)	F12 (M)		A109(660)		W11 (M)
L42(593)	F12 (M)		N110(661)		W11 (M)
F43(594)	W11 (H,S,M)		V111(662)		F12 (M)
M44(595)	W11 (M)		Q112(663)		F12 (M)
A45(596)	W11 (ကMက)		G113(664)		W11 (M)
M46(597)	W11 (ကMက)		L114(665)		Y25 (M)
E47(598)	W11 (ကMက)	K28 (H)	S115(666)		Y25 (H, M)
Y49(600)		R29 (H) I33 (M)	R118(669)		I27 (M)
M51(602)	W11 (ကMက)		S119(670)		I27 (M) N30 (H)
E53(604)	K24 (I)	L21 (M)			
H54(605)	Y25 (M)				
F55(606)	Y25 (M)				
D56(607)		K24 (I,H) V35 (M)			
N57(608)	Y25 (H,M)				
V58(609)	Y25 (M)				
L59(610)	W11 (ကMက)				

a H, T, I, and M denote the types of interaction: hydrogen bond, T-shaped stacking, ionic bond, and molecular hydrophobicity potential contact (Efremov et al., 2007), respectively.

b Larger values of ZDock score correspond to a more energetically favorable complex.

In the second model (**Figure 9C**, **Table 1**, #2), the toxin is located on the top of the domain, and its β-hairpin penetrates into the cleft formed by the S1-S2 and S3-S4 loops. This complex is stabilized by the ionic bridge between charged groups of K24 and D56(607) and by hydrogen bonds with residue E47(598) (**Table 1**). Despite the larger VSD-II/Hm-3 contact area in this complex (∼370 vs ∼290 Å^2^), it has lower value of ZDock score (1,077.8 vs 1,098.7), which corresponds to less energetically favorable complex (**Table 1**). Moreover, model #2 does not fully agree with the experimental NMR data. Aromatic groups of the toxin residues W11 and F12 form tight contacts with residue V111(662) in the VSD-II S3-S4 loop, for which no changes in NMR parameters were observed (**Figures 8A, B**). In addition, the tip of the toxin β-hairpin overlaps significantly with the pore domain (**Figure 9C**, #2). Nevertheless, such mode of Hm-3 binding to the S1-S2 loop is probably possible in the down-state of VSD-II (see later discussion).

## Discussion

### Cell-Free Expression Platform for Nuclear Magnetic Resonance Studies of Ligand–Receptor Interactions

NMR structural studies of proteins depend on the recombinant production of samples labeled with stable isotopes (^2^H, ^13^C, ^15^N). CF protein expression systems provide a cheap alternative to the host-based expression (Maslennikov et al., 2010). Use of individual ^13^C- and ^15^N-labeled AAs allows production of protein samples selectively labeled by certain AA types, while totally ^13^C,^15^N-labeled proteins can be easily produced using algal AA mixtures supplied with missing AAs (Trp, Gln, and Asn). NMR studies of membrane proteins require not only efficient expression but also selection of membrane-mimicking media for protein stabilization. In case of ligand–receptor interaction studies, the relevant media should preserve the quasi-native structure of the receptor and should not prevent binding of the ligands. These properties of membrane-mimicking media frequently do not correlate with the quality of the NMR spectra (Shenkarev et al., 2010). The results of present investigation indicate that DPC and DPC/LDAO micelles preserve the ability of VSD-II to interact with Hm-3 toxin (**Figure 4A** and **S1**). Nevertheless, the quality of NMR spectra in these media was not sufficient for the classic structure determination protocols.

In the present report, we describe an alternative approach for the structural studies of ligand–receptor interactions, which relies on the CF synthesis of selectively ^13^C,^15^N-labeled protein samples. The special combinatorial pattern of the isotope labels incorporation allowed us to assign ∼50% of the VSD-II backbone resonances (**Figure 8A**). This was sufficient for a characterization of the secondary structure and ps–ns timescale dynamics of the VSD-II molecule (**Figure 5**), for mapping of the toxin–domain interaction interfaces (**Figures 7D** and **8B**) and modeling of the VSD-II/Hm-3 complexes (**Figure 9**). In addition, the EPR data obtained for double spin-labeled VSD-II confirmed the quasi-native domain fold in the micelles (**Figure 6**).

We propose that the described framework is generally applicable for NMR studies of the ligand–receptor interactions in moderately sized (up to 200 AAs) membrane proteins. Studies of larger IMPs will require extensive side-chain deuteration (^2^H-labeling), which can also be done in CF systems (Etezady-Esfarjani et al., 2007). The general applicability of the proposed approach is illustrated by a recent study of the Hm-3 complex with VSD-I of Na_V_1.4 channel (Männikkö et al., 2018). Samples of this domain had extremely low stability in micellar environment (half-life of ∼24 h). Nevertheless, our method allowed us to acquire sufficient data about the interaction interfaces in the complex.

### S1-S2 Loop Is an Alternative Binding Site for Gating Modifier Toxins

Despite the rapid progress in structural studies of voltage-gated channels achieved during the last decade, little is known about the mechanism of spider GMTs interaction with Na_V_ and K_V_ channels. The pioneering work of Swartz and MacKinnon revealed that the outer fragment of the VSD S3 helix (S3b) is the major binding site of hanatoxin to the K_V_2.1 channel (**Figure 1C**) (Swartz and MacKinnon, 1997). Recent mutagenesis (Bosmans et al., 2008) and structural data obtained for the complex of Na_V_1.4 VSD-I with Hm-3 toxin (Männikkö et al., 2018) and complex of Na_V_AB/hNa_V_1.7-DII chimera with ProTx2 (Xu et al., 2019) confirmed that the S3b helix is the main interaction site in Na_V_/GMT complexes. In the previously mentioned channels, the S3b helix contains one or two negatively charged Asp/Glu residues (**Figure 1C**, blue boxes), which form ionic bridges with the Lys/Arg residues located in the toxin β-hairpins (Xu et al., 2019). It should be noted that in VSD-I of Na_V_1.4 channel, the corresponding charged groups (E208/D211) are separated by two residues.

Electrophysiology data obtained previously (Männikkö et al., 2018) and in the present work (**Figure 2**) suggest that Hm-3 is able to interact not only with VSD-I but also with VSD-II of Na_V_1.4 channel. At the same time, this interaction probably relies on a different mechanism. First, Hm-3 inhibits gating pore currents due to mutations of the R2 or R3 residues in the S4 helix of VSD-I, while in VSD-II, it inhibits only currents associated with the R3 mutation. Second, Hm-3 efficiently inhibits voltage activation of the chimeric K_V_2.1 channel with the S3-S4 paddle motif transferred from VSD-I of Na_V_1.4 but does not significantly influence activation of the K_V_2.1/VSD-II chimera. The obtained NMR data explain the observed differences. It was found that Hm-3 targets a different site in VSD-II. This site is formed by the outer fragments of the S1 and S2 helices and the S1-S2 extracellular loop and contains three negatively charged residues [S1: E47(598), S1-S2: E53(604), and S2: D56(607)], which may potentially form ionic bridges with the toxin. Further electrophysiological studies are required to experimentally confirm the interaction of these residues with Hm-3.

The binding site on the Hm-3 surface is surprisingly identical for both VSD-I and VSD-II and comprises the N-terminal strand and the tip of the β-hairpin with three positively charged groups (K24, K28, and R29). Thus, we propose that the Hm-3 β-hairpin is optimized to interact with helical structures containing two acidic groups separated by two or more residues [e.g., E208/D211 in VSD-I; and E53(604)/D56(607) in VSD-II]. This proposal explains why Hm-3 targets the S1-S2 loop in VSD-II. Indeed, the S3-S4 loop of this domain does not have the required spatial signature and contains only one charged group E105(656) directed toward the interior of the four-helix VSD bundle (**Figure 8B**).

Na_V_1.4 is not a unique channel for which GMT binding to the S1-S2 loop has been described. Previously, such binding mode has been observed by NMR in the complex of VsTx1 toxin with the VSD of archaeal K_V_AP channel. The β-hairpin of this toxin protrudes into the cleft between the S1 and S4 helices, forming an ionic bridge with residue E45 from the S1 helix [homologous to E47(598) in Na_V_1.4 VSD-II] (Lau et al., 2016). Another binding mode was observed in the cryo-EM structure of the insect Na_V_PaS channel in complex with the Dc1a toxin (Shen et al., 2018). The elongated β-hairpin of Dc1a protrudes into the cavity between the S1-S2 and S3-S4 loops in VSD-II and forms ionic bridges with residues D539/D542 of the S1-S2 loop [D539 is homologous to E47(598) in Na_V_1.4 VSD-II]. Interestingly, the tip of Dc1a β-hairpin contacts a fragment of the third repeat participating in the formation of the channel pore domain.

Spider GMTs are well known for their ability to interact with multiple targets (Redaelli et al., 2010). The results obtained here indicate that one toxin may target not only several subtypes of Na_V_ and K_V_ channels but may also have multiple binding sites within one Na_V_ molecule. Surprisingly, these sites located at different VSDs can involve structurally different interfaces. Thus, we may speculate not only about “target promiscuity” of spider toxins (Redaelli et al., 2010) but also about “domain or binding site promiscuity” within one channel.

### Possible Mechanism of Gating Pore Current Inhibition by Hm-3

Alterations in the TM potential induce conformational changes in VSDs, which in turn lead to gating of the channel pore. Depending on the TM potential value, VSDs can adopt a set of conformations, which differ by the position of the S4 helix relative to the membrane and other TM helices of the VSDs [up, down, and some intermediate states (Tao et al., 2010; Henrion et al., 2012; Jensen et al., 2012)]. The gating pore currents through VSDs are also state-dependent, and they arise when a mutated gating charge residue from the S4 helix contacts with the conserved residues of the hydrophobic gating charge transfer center (formed by residues from the S1 and S2 helices). The recent crystal structure of the Na_V_Ab(R3G) mutant channel showed that, in this case, a continuous water-accessible path through the VSD is formed (Jiang et al., 2018).

Hm-3 inhibits activation of Na_V_1.4 channel and blocks depolarization-activated gating pore currents in the R3 mutants of VSD-I and VSD-II. This suggests that the toxin interacts with and stabilizes the down-state of VSDs, preventing depolarization-activated structural transition to the up-state. However, our NMR and molecular modeling data (obtained in the absence of voltage) describe Hm-3 interaction with the up-state of VSD-II. Probably, similar to other GMTs from spider venom (Phillips et al., 2005), Hm-3 interacts with both up- and down-states of VSD-I and VSD-II but has a higher affinity to the down-state. The fact that Hm-3 inhibits gating pore currents in the R2 mutant of VSD-I, but not of VSD-II, illustrates the higher affinity of the toxin to the “down”-state of VSD-I or is a result of different binding conformations of Hm-3 to distinct VSDs. It is unclear why Hm-3 does not change the voltage dependence of the DII-R2G mutant.

Protein–protein docking guided by NMR restraints predicted two possible modes of Hm-3 interaction with the up-state of VSD-II. In the first case (**Figure 9C**, model #1), the toxin binds to the membrane-exposed side of the S1-S2 loop at the periphery of the domain. This structure resembles Na_V_1.7-DII/ProTx2 and VSD-I/Hm-3 complexes, which are additionally stabilized by toxin–membrane interactions. The contacts of W11, F12, and W16 residues of Hm-3 with the membrane surrounding the domain are in line with the proposed membrane-mediated mechanism of GMT action (Milescu et al., 2007). Interestingly, this mode of VSD-II/Hm-3 interaction is not sensitive to the VSD state, since vertical movement of the S4 helix does not significantly influence the S1-S2 loop conformation (Clairfeuille et al., 2019).

In the second model (**Figure 9C**, #2), Hm-3 molecule tightly associates with both the S1-S2 and S3-S4 loops approaching VSD-II from the extracellular side and contacting the pore region of the third repeat by the tip of the β-hairpin. This model resembles the Na_V_PaS/Dc1a complex (see previous discussion), where no pronounced toxin–membrane interactions were observed. At the same time, this mode of toxin binding can considerably change upon VSD switching to the down-state, where toxin–membrane interactions can be restored. The present data do not permit an unequivocal selection of the best model of Hm-3 interaction with a physiologically relevant down-state of VSD-II. Additional computer simulations are required to solve this controversy. Nevertheless, both possible models of the VSD-II/Hm-3 complex have one common feature: the bound toxin molecule disrupts ionic interactions stabilizing the up-state of VSD-II.

The side chains of E47(598) and D56(607) in VSD-II stabilize the first gating charge R1 [R118(669)] residue in a position close to the extracellular side of the membrane. After minimal adaptation of Hm-3 and VSD-II conformations in the complex, the positively charged toxin groups may disrupt these intra-VSD interactions. K28 and K24 side chains of Hm3 (models #1 and #2, respectively) may form an ionic bridge with residue D56(607) and thereby destabilize the up-state of the S4 helix. The observed significant changes in the NMR signals of residues R118(669), N40(591), T41(592), and G68(619) (**Figure 8C**) confirm this assumption.

Our results indicate that prevention of voltage sensor movement to the active up-state can reduce the depolarization-activated gating pore currents in mutant channels found in patients with NormoPP. However, as this also results in the inhibition of channel opening, these toxins do not serve as optimal hits for NormoPP therapy. We propose that the destabilization of the ionic interactions on the extracellular face of VSDs of Na_V_1.4 channel by domain-selective compounds may induce allosteric changes in the S4 helix resulting in the inhibition of the depolarization-activated gating pore currents. Modifications of toxin structure can bring about drastic effects on its affinity and selectivity to different Na_V_s (Park et al., 2014; Murray et al., 2016). Structural studies such as described here, in combination with screening of mutant channels and toxins, may help develop selective inhibitors of gating pore currents relative to main pore currents and of gating pore mutant channels relative to the wild-type channel.

## Data Availability

All datasets generated for this study are included in the manuscript and/or the **Supplementary files**.

## Author Contributions

MM and AP performed NMR experiments. RM performed and analyzed Na_V_1.4 electrophysiology experiments. OK and MF performed EPR experiments. DSK produced recombinant VSD-II. AC performed molecular modeling, AB and MS prepared toxin samples. MH and DMK supervised electrophysiology experiments. EB supervised EPR experiments. AA and ZS supervised NMR experiments. EL designed and supervised the CF production. MK supervised the bioengineering. MM, RM, OK, AV, and ZS designed the study, analyzed the data, and wrote the manuscript.

## Funding

The work was supported by the Russian Science Foundation (project № 16-14-10338). Computer modeling and Hm-3 production were performed with the financial support of the Russian Academy of Sciences (Program “Molecular and Cell Biology”). *Xenopus* oocyte work was supported by MRC project grant (MR/M006948/1). OK and EB are thankful to the Ministry of Science and Education of the Russian Federation (grant 14.W03.31.0034) for financial support of the EPR measurements.

## Conflict of Interest Statement

The authors declare that the research was conducted in the absence of any commercial or financial relationships that could be construed as a potential conflict of interest.
