# Supplementary material for: Cell-Free Expression of Sodium Channel Domains for Pharmacology Studies. Noncanonical Spider Toxin Binding Site in the Second Voltage-Sensing Domain of Human Nav1.4 Channel
Source: Front Pharmacol. 2019 Sep 4;10:953. doi: 10.3389/fphar.2019.00953 (PMC6737007; doi:10.3389/fphar.2019.00953)
Supplement: Supplementary file 1 [file DataSheet_1.doc]

**Supporting Information**

**Cell-Free Expression of Sodium Channel Domains for Pharmacology Studies. Noncanonical Spider Toxin Binding Site in the Second Voltage-Sensing Domain of Human Na_V_1.4 Channel**

Mikhail Yu. Myshkin^1^, Roope Männikkö^2^, Olesya A. Krumkacheva^3^, Dmitrii S. Kulbatskii^1^, Anton O. Chugunov^1,4,5^, Antonina A. Berkut^1^, Alexander S. Paramonov^1^, Mikhail A. Shulepko^1^, Matvey V. Fedin^3^, Michael G Hanna^2^, Dimitri M. Kullmann^6^, Elena G. Bagryanskaya^7^, Alexander S. Arseniev^1,4^, Mikhail P. Kirpichnikov^1,8^, Ekaterina N. Lyukmanova^1,4^, Alexander A. Vassilevski^1,4^, Zakhar O. Shenkarev^1,4*^

*^1^Shemyakin and Ovchinnikov Institute of Bioorganic Chemistry, Russian Academy of Sciences, Miklukho-Maklaya str., 16/10, Moscow, 117997, Russia;*

^2^MRC Centre for Neuromuscular Diseases, Department of Molecular Neuroscience, UCL Institute of Neurology, WC1N 3BG London, United Kingdom;

^3^International Tomography Center SB RAS, Institutskaya 3a, Novosibirsk 630090, Russiа;

^4^Moscow Institute of Physics and Technology (State University), Institutskiy Pereulok 9, Dolgoprudny, Moscow Region, 141700, Russia;

^5^National Research University Higher School of Economics, Moscow, 101000, Russia;

^6^Department of Clinical and Experimental Epilepsy, UCL Institute of Neurology, WC1N 3BG London, United Kingdom;

^7^N.N.Voroztsov Novosibirsk Institute of Organic Chemistry SB RAS, Pr. Lavrentjeva 9, Novosibirsk 630090, Russia

^8^Lomonosov Moscow State University, Moscow, 119991, Russia;

Correspondence to Zakhar O. Shenkarev [zakhar-shenkarev@yandex.ru](mailto:zakhar-shenkarev@yandex.ru)


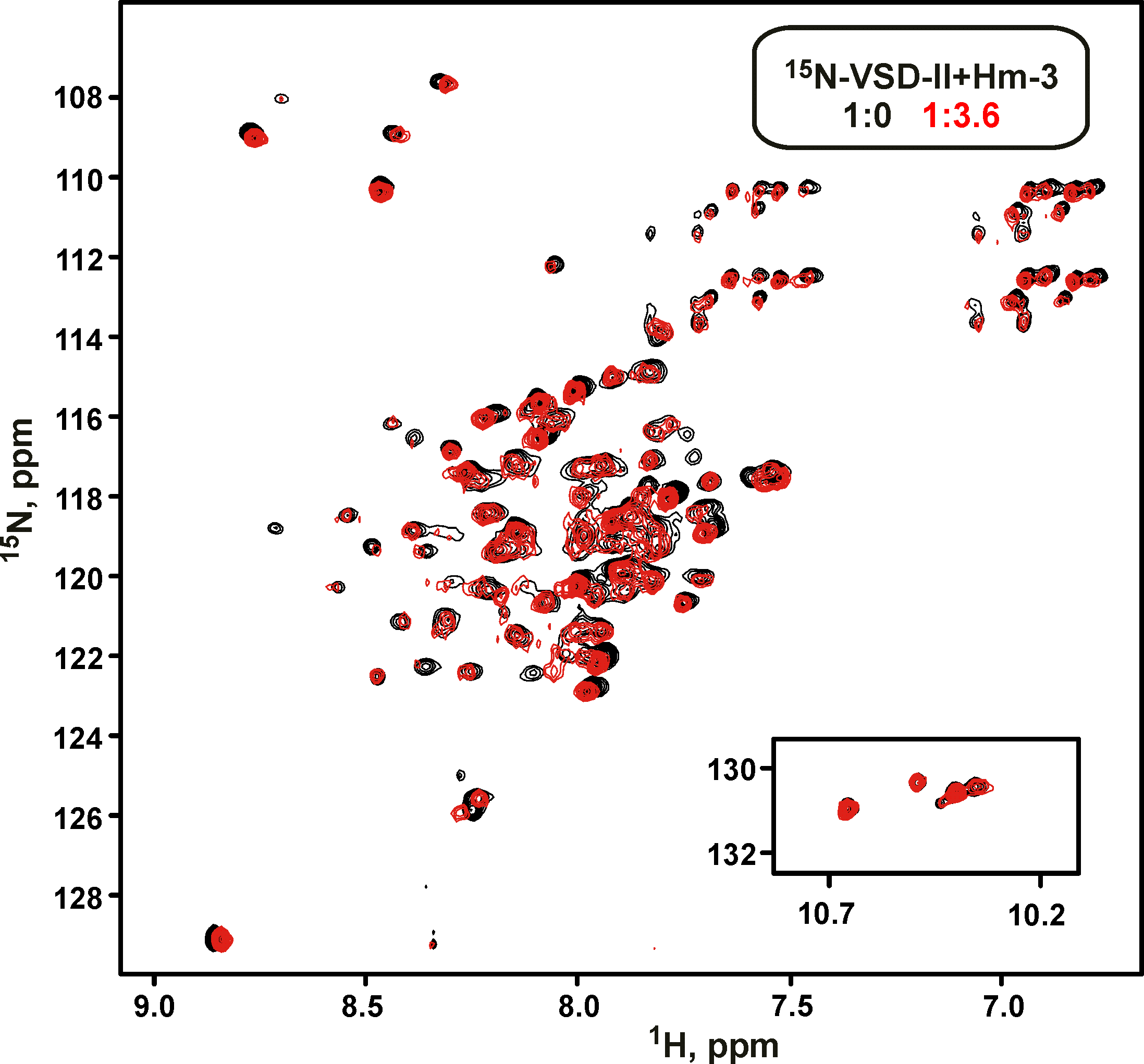


FIGURE S1. Overlay of ^1^H,^15^N-TROSY spectra of 100 μM ^15^N-labeled VSD-II in DPC (90 mM, pH 5.0, 45 °C, 800 MHz) before (black) and after (red) addition of 360 μM unlabeled Hm-3. The insert shows ^1^H^15^N^ε1^ signals of Trp side chains.


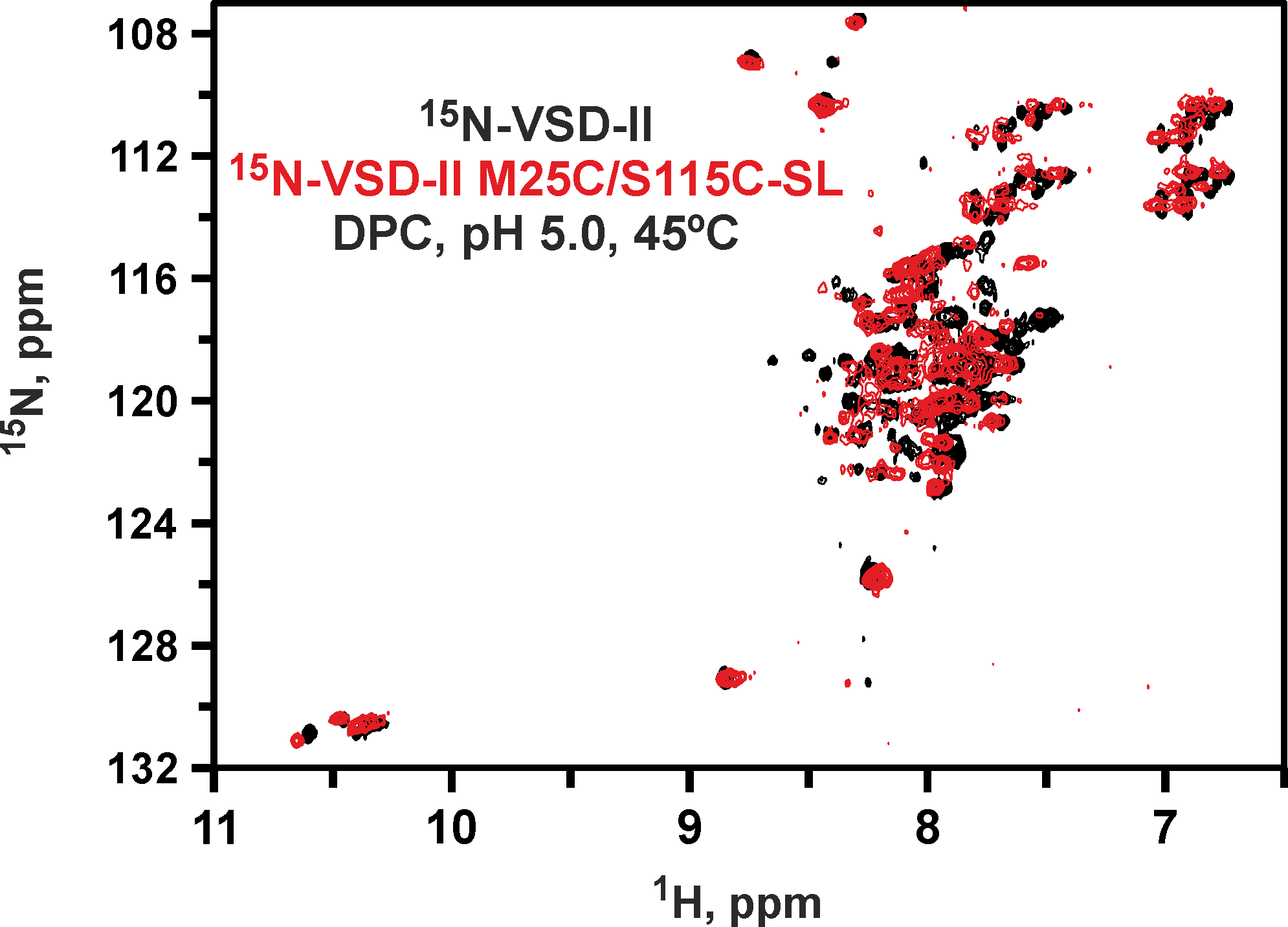


FIGURE S2. Overlay of ^1^H,^15^N-TROSY spectra of the double ^15^N/MTSL-labeled M25/S115 VSD-II in DPC micelles (red) compared with the spectrum of ^15^N-VSD-II (black).


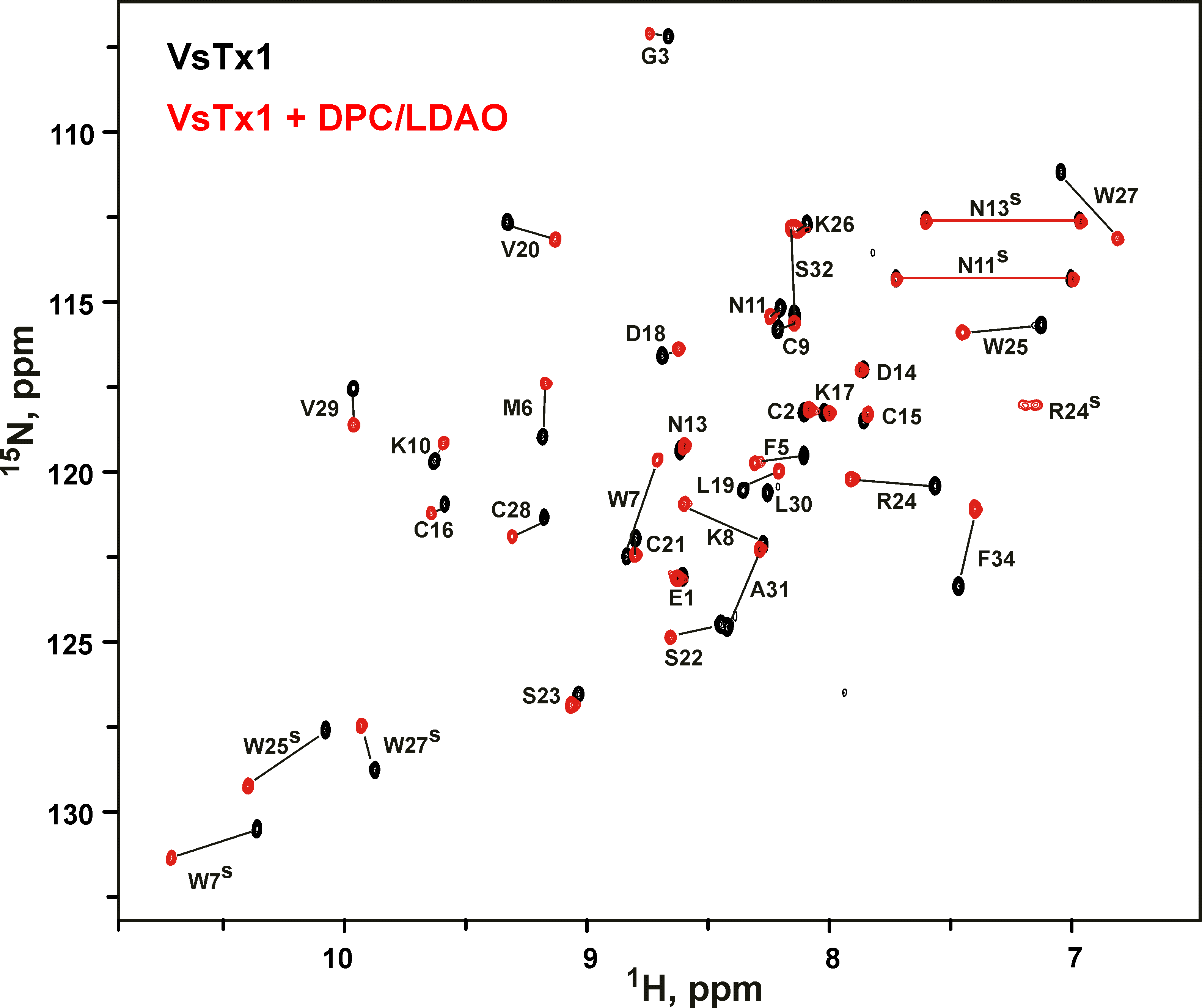


FIGURE S3. Overlay of ^1^H,^15^N-HSQC spectra of 30 μM ^15^N-labeled VsTx1 before (black) and after (red) addition of 45 mM DPC and 45 mM LDAO (pH 5.5, 45 °C, 800 MHz).
